# Reference genomes of the two cultivated jute species

**DOI:** 10.1111/pbi.13652

**Published:** 2021-07-08

**Authors:** Lilan Zhang, Xiaokai Ma, Xingtan Zhang, Yi Xu, Aminu Kurawa Ibrahim, Jiayu Yao, Huaxing Huang, Shuai Chen, Zhenyang Liao, Qing Zhang, Sylvain Niyitanga, Jiaxin Yu, Yi Liu, Xiuming Xu, Jingjing Wang, Aifen Tao, Jiantang Xu, Siyuan Chen, Xin Yang, Qingyao He, Lihui Lin, Pingping Fang, Liemei Zhang, Ray Ming, Jianmin Qi, Liwu Zhang

**Affiliations:** ^1^ Key Laboratory of Ministry of Education for Genetics, Breeding and Multiple Utilization of Crops Fujian Provincial Key Laboratory of Crop Breeding by Design Fujian Agriculture and Forestry University Fuzhou China; ^2^ Experiment Station of Ministry of Agriculture and Rural Affairs for Jute and Kenaf in Southeast China Fujian Public Platform for Germplasm Resources of Bast Fibre Crops Fujian International Science and Technology Cooperation Base for Genetics, Breeding and Multiple Utilization Development of Southern Economic Crops Fujian Agriculture and Forestry University Fuzhou China; ^3^ Center for Genomics and Biotechnology Haixia Institue of Science and Technology Fujian Agriculture and Forestry University Fuzhou China; ^4^ Department of Plant Biology the University of Illinois at Urbana‐Champaign Urbana IL USA

**Keywords:** jute (*Corchorus*), genome, artificial selection, bast fibre, domestication

## Abstract

Cultivated jute, which comprises the two species *Corchorus capsularis* and *C. olitorius*, is the second most important natural fibre source after cotton. Here we describe chromosome‐level assemblies of the genomes of both cultivated species. The *C. capsularis* and *C. olitorius* assemblies are each comprised of seven pseudo‐chromosomes, with the *C. capsularis* assembly consisting of 336 Mb with 25,874 genes and the *C. olitorius* assembly containing 361 Mb with 28 479 genes. Although the two *Corchorus* genomes exhibit collinearity, the genome of *C. olitorius* contains 25 Mb of additional sequences than that of *C. capsularis* with 13 putative inversions, which might give a hint to the difference of phenotypic variants between the two cultivated jute species. Analysis of gene expression in isolated fibre tissues reveals candidate genes involved in fibre development. Our analysis of the population structures of 242 cultivars from *C. capsularis* and 57 cultivars from *C. olitorius* by whole‐genome resequencing resulted in post‐domestication bottlenecks occurred ~2000 years ago in these species. We identified hundreds of putative significant marker‐trait associations (MTAs) controlling fibre fineness, cellulose content and lignin content of fibre by integrating data from genome‐wide association studies (GWAS) with data from analyses of selective sweeps due to natural and artificial selection in these two jute species. Among them, we further validated that *CcCOBRA1* and *CcC4H1* regulate fibre quality in transgenic plants via improving the biosynthesis of the secondary cell wall. Our results yielded important new resources for functional genomics research and genetic improvement in jute and allied fibre crops.

## Introduction

Bast (phloem) fibres are extracted mainly from the stems of jute, kenaf, flax, ramie, hemp and other plant species. Jute is one of the most important natural bast fibre crops and belongs to the genus *Corchorus*, which includes more than 100 species. Two diploid species (2*n* = 2*x* = 14), white jute (*C. capsularis*) and dark or tossa jute (*C. olitorius*), are cultivated for white fibre and golden fibre, respectively. The cultivated species differ in flower characteristics, silique shape and seed color. *C. capsularis* and *C. olitorius* are not sexually compatible (i.e. they do not form fertile hybrids).

India, Bangladesh and China are the world’s major suppliers of jute fibre. The annual global production of jute fibre is 3 422 876 tonnes with a value of 2.3 billion USD (http://www.fao.org/). The history of jute cultivation in China is long and was first recorded in the *Tu Jing Ben Cao* (1061 AD). Jute is cultivated as a raw material to produce many diverse textile goods, such as coarse cloth, paper, rope, canvas and burlap. *C. capsularis* was probably domesticated in Indo‐Myanmar or possibly originated in Africa, but was domesticated in Asia, while *C. olitorius* had its origin in equatorial region of east Africa, but was domesticated in India and southern China (Kundu *et al*., 2013).

Draft genomes of *C. capsularis* and *C. olitorius* were previously generated using short reads from next‐generation sequencing (NGS) (Islam *et al*., 2017; Sarkar *et al*., 2017). Although these draft genomes enabled genomic research in *C. capsularis* and *C. olitorius* to progress, the initial assemblies were composed of contigs that were often arbitrarily ordered and oriented. In contrast, the chromosome‐level reference genomes for jute will provide fundamental tools for the study of domestication and population genetics. Further, these improved genomic resources could also pave the way to further analyses of the mechanistic basis of various economically and biologically important traits in jute.

Cellulose content, lignin content and fibre fineness are all important traits that affected bast fibre quality, and they are the main determinants of fibre quality improvement in the future. By regulating the expression of key genes of lignin synthesis (such as *C4H* and *PAL*), it was found that the lignin content of transgenic plants was significantly reduced and the fibre quality was significantly improved (Zhang *et al*., 2020a). In addition, it is of great significance to study secondary cell wall (SCW) biosynthesis and promote the innovation of germplasm resources to study the expression and function of genes related to SCW and fibre quality. Transcription factors, such as MYB, WRKY and NAC, play an important role in the regulatory network of SCW biosynthesis (Chakraborty *et al*., 2015; Samanta *et al*., 2015; Satya *et al*., 2018; Yang *et al*., 2020). They can improve the fibre quality of plants by activating or inhibiting the expression of downstream genes.

Many quantitative trait loci (QTL) related to important traits have recently been tagged using molecular markers in populations of jute (Das *et al*., 2012; Kundu *et al*., 2015; Tao *et al*., 2017). Association mapping of important traits with molecular markers has also been performed in jute, and some markers associated with important traits have been detected. However, these studies were limited by a relatively low number of polymorphic markers; thus, important candidate genes had not yet been identified using such map‐based approaches. However, with the rapid development of DNA sequencing technologies in recent years, genome‐wide association analysis (GWAS) has overcome the earlier limitations of QTL mapping and association analysis, and many loci related to important target traits in many crops have now been mapped (Cavanagh *et al*., 2013; Korte and Farlow, 2013; Swarts *et al*., 2017; Zhou *et al*., 2017).

Here we present new chromosome‐level reference genomes for both *C. capsularis* and *C. olitorus*. Additionally, we describe a high‐density single‐nucleotide polymorphism (SNP) marker map for jute. The reference genomes will serve as fundamental resources for studies of evolution and domestication of jute while enabling population genetic research. The SNP marker map will provide an essential resource for GWAS in jute and will facilitate the detection of genetic changes associated with jute divergence, domestication and improvement. During the process of domestication and modern breeding, jute has undergone strong and extensive artificial selection for fibre quality. Genes that are under artificial selection are usually associated with fibre quality‐related traits. Thus, a better understanding of the details of genetic variation and identification of selective sweeps during crop improvement can provide guidelines for further crop improvement. However, the impact of selection on the patterns of genetic variation in jute remains largely unknown so far.

## Results

### Assembly, annotation and phylogenetic analysis

**Figure 1:**
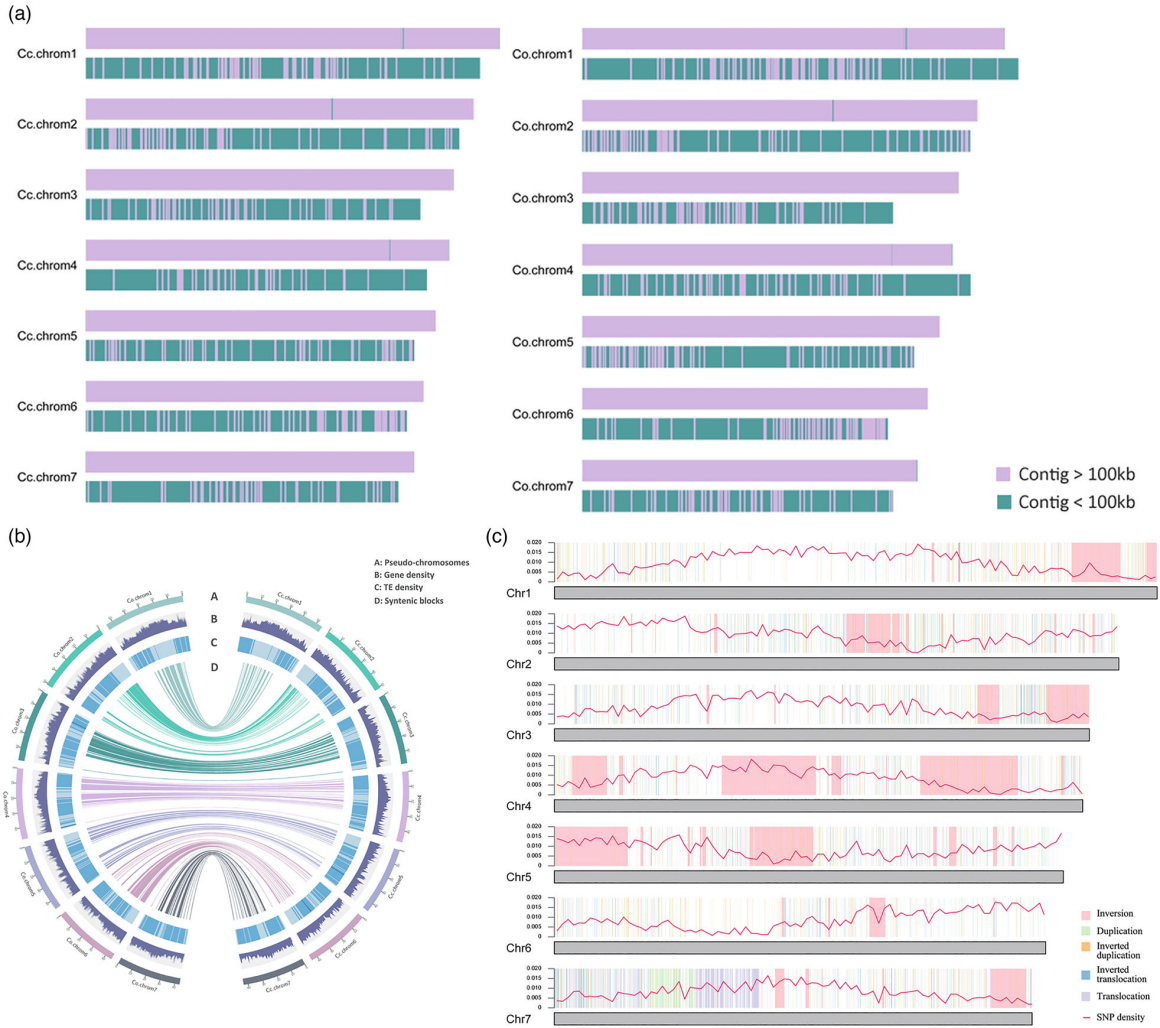
Genome assembly and phylogenetic analysis of *Corchorus capsularis* and *C. olitorius*. (a) Genome assembly layout. Comparison of *C. capsularis* var. ‘Huangma 179’ (HM179) and *C. olitorius* var. ‘Kuanyechangguo’ (KYCG) reference genomes contig level assembly with the previously published genomes contig level assembly. The top track shows the present genomes contig level assembly, while the bottom track shows the previously published genomes contig level assembly. (b) Basic genome information, including pseudo‐chromosomes (A), transposable elements density (B), gene density (C) and syntenic block (D), in *C. capsularis* and *C. olitorius*. (c) Genome‐wide distribution of the structural variations identified between *C. capsularis* and *C. olitorius*.

We sequenced the genomes of *C. capsularis* var. ‘Huangma 179’ (HM179) and *C. olitorius* var. ‘Kuanyechangguo’ (KYCG) (Figure 1b; Figure S1). The genome size was estimated to be 348 Mb for *C. capsularis* and 387 Mb for *C. olitorius* by 19 K‐mer analysis based on high‐quality Illumina reads (Figure S2). Approximately 41 Gb (~120× coverage) and 41 Gb (~112× coverage) of raw single molecule real‐time (SMRT) data were generated using PacBio Sequel for the two species, respectively (Table S1). The contig‐level assemblies were constructed using the CANU package (Koren *et al*., 2017). Hi‐C libraries yielded 149 million clean paired‐end reads for *C. capsularis* and 240 million clean paired‐end reads for *C. olitorius* (Table S2). These paired‐end Hi‐C reads were then uniquely mapped onto contigs, which were grouped into seven pseudo‐chromosomes in each species (Figure 1b; Figure S3; Table S3). The resulting assemblies contained 336 Mbp for *C. capsularis* with an N50 of 46 Mb and 361 Mbp for *C. olitorius* with an N50 of 50 Mb, showing an extensively improved assembly compared to previously published jute genomes (Islam *et al*., 2017) (Table 1).

**Table 1:** Global statistics comparison of *Corchorus capsularis* and *C. olitorius* assembly between the present genomes and previously published genomes

Items	Previously published genomes		Present genomes			
	Contig level assembly		Contig level assembly		Chromosomal level assembly	
	*C. capsularis*	*C. olitorius*	*C. capsularis*	*C. olitorius*	*C. capsularis*	*C. olitorius*
No. of sequences	16,522	24,918	340	678	71	284
Max length (Mb)	0.34	0.36	17	15	57	60
Total size (Mb)	317	335	340	394	336	361
N50 (Mb)	0.046	0.045	3,2	1,5	46	50
Average length (bp)	19,197	13,440	1,001,680	580,535	4,797,158	1,386,532

To validate our assembly, both genomes were compared with the previously published draft genomes (Islam *et al*., 2017), showing two vastly improved *de novo* assembly and annotation of the jute reference genomes (Figure 1a). The detection by BUSCO (Benchmarking Universal Single‐Copy Orthologs) of 2191 (94.2%) BUSCOs in the *C. capsularis* assembly and 2195 (94.4%) in the *C. olitorius* assembly out of the expected 2326 complete BUSCOs (Table S4) reflects improvement of these jute genome assemblies compared to earlier ones. The BUSCO analysis showed 86.1% and 90.8% of gene models detected were complete (as opposed to fragmented or missing) in the assembled genome sequences of *C. capsularis* and *C. olitorius*, respectively (Table S5). Compared with the previously published draft genomes, these sequences represent a significant improvement in integrity and contiguity. These new genome sequences will serve as reference assemblies for both *C. capsularis* (white jute) and *C. olitorus* (dark jute). Further, during our CEGMA analysis (Parra *et al*., 2007) (Table S6) of these genome assemblies, we could predict 235 (94.76%) gene models in the *C. capsularis* assembly and 240 (96.77%) in the *C. olitorius* assembly out of the 248 expected ultra‐conserved core eukaryotic genes (CEGs), which indicates the high quality of our assemblies.

Using a combination of *de novo* gene prediction, protein homology searches and assembly of RNA‐Seq reads, 25,874 genes were predicted in the *C. capsularis* genome and 28,479 genes were predicted in the *C. olitorius* genome. The average gene lengths and the average numbers of exons in *C. capsularis* genes are 3933 bp and 6.5, respectively, while those in *C. olitorius* are 3886 bp and 6.3, respectively (Table S7). The percentages of total repeat fraction in the *C. capsularis* and *C. olitorius* genomes were 53.6% and 59.33%, respectively (Table S8), which all indicates that these assemblies could serve as reference genomes for jute.

The high contiguity and completeness of the genome assemblies represent major improvements in regions with high contents of repeat sequences. We observed the two closely related species had different LTR burst events. It is estimated that the LTR insertion occurred at 0.6 mya in *Cc*, while *Co* showed a more recently LTR burst, dating back to 0.4 mya (Figure S4).

We conducted phylogenetic analyses of the genomes of *C. capsularis* and *C. olitorius* compared to other genomes in Malvaceae. By calculating nonsynonymous (Ka) and synonymous (Ks) substitution rates for orthologous gene pairs, we propose that the speciation between *Corchorus* and *G. raimondii* occurred ~38 MYA (million years ago) and that between *Corchorus* and *T. cacao* occurred ~16.75 MYA (Table S9). The divergence between *C. capsularis* and *C. olitorius* likely occurred ~4.63 MYA (Figure S5; Table S9).

The high‐quality reference genomes allowed us to identify large structural variants by a direct comparative genomic analysis of the two species (Figure 1a). High collinearity was observed between *C. capsularis* and *C. olitorius* genomes, although the genome of *C. capsularis* was 25 Mb smaller than that of *C. olitorius* (Table 1; Figure S6). Through computational comparisons of these genome sequences, the current study identified 13 putative inversions that were all larger than 1 Mb (Figure 1c). Our GO term enrichment analysis revealed that the genes within these inversions were significantly enriched for GO terms related to ‘external encapsulating structure’, ‘plastid stroma’, and ‘chromoplast stroma’, ‘cell wall’ (*P* < 0.05, Fisher’s exact test) (Figures S7 and S8; Table S10).

### Transcriptome analysis and analysis of genes involved in fibre development

In general, there are significant differences in fibre quality traits between the two cultivated jute species. Based on the two chromosome‐level jute genomes, we compared the expression of transcripts and genes that are related to fibre development in the secondary cell wall with phytohormone, especially gibberellin. Gibberellin, the key plant hormone associated with the reduction of plant height by plant breeders during the Green Revolution in the middle of the 20th century, and the main function of which is to promote cell elongation (Honi *et al*., 2020). Thus, transcriptome analysis of stem bark grown under either normal conditions or after GA_3_ treatment at the vigorous vegetative stage (60 days after germination) in HM179 and KYCG was conducted to identify candidate genes that are involved in fibre development of bast fiber (Table S11).

**Figure 2:**
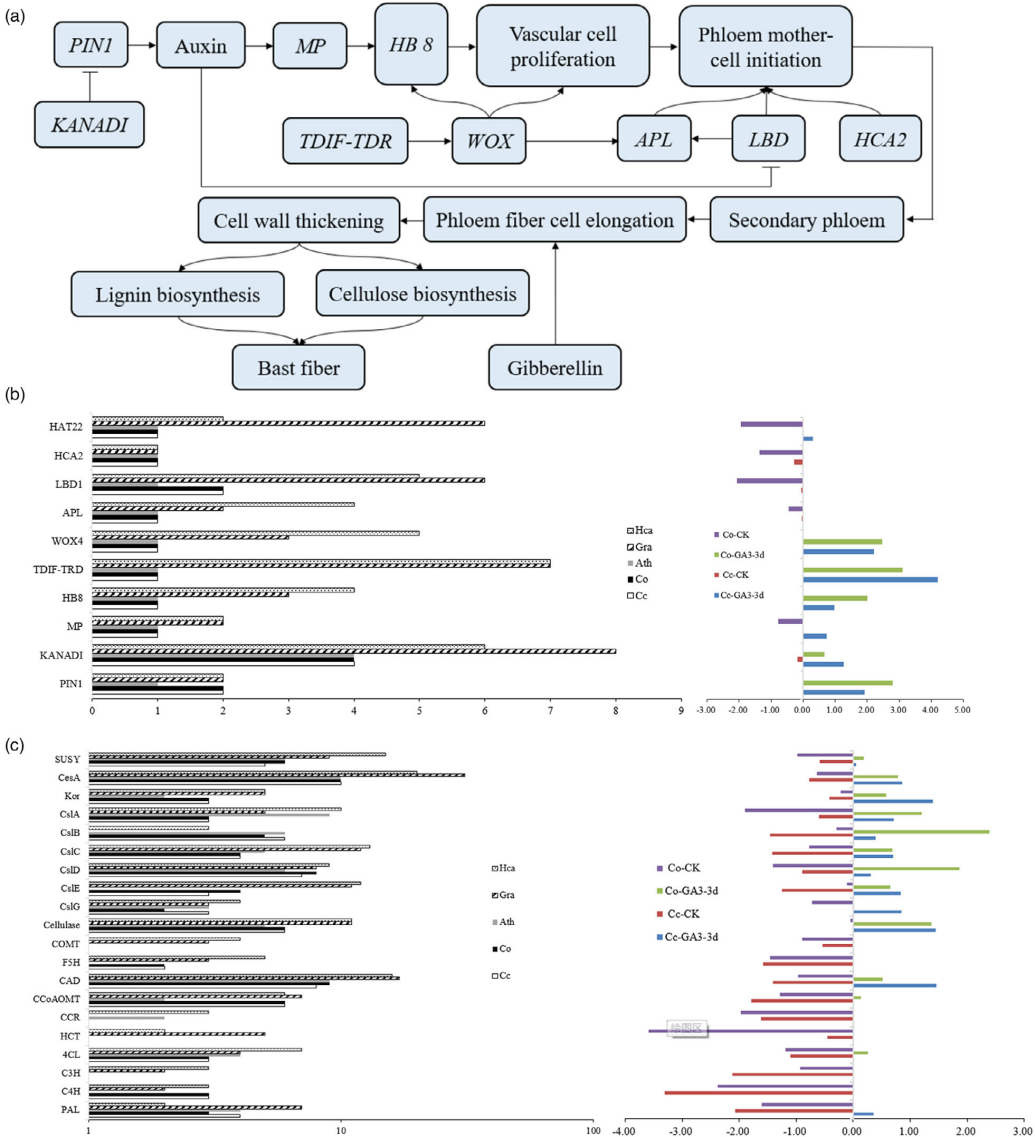
Overview of fibre development regulation in jute (*Corchorus capsularis* and *C. olitorius*). (a) Schematic map of fibre formation. Fibre formation related genes are listed in Table S11. (b) Comparison of copy numbers and expression of genes involved in fibre initiation and elongation among *Cc*, *Co*, *Ath, Gra* and *Hca*. On the left panel, *x*‐axis indicates number of genes and y‐axis presents name of target genes. Expression profile of genes involved in fibre initiation and elongation in stem barks between control (CK) and 3 days after GA_3_ treatment (GA_3_.3d) at the vigorous growth stage is shown on the right panel. *Cc*, *C. capsularis*; *Co*, *C. olitorius*; *Ath*, *A. thaliana*; *Gra, G. raimondii*; *Hca, H. cannabinus*. (c) Comparison of copy numbers (left panel) and expression (right panel) of genes involved in lignocellulosic biosynthesis enzymes among *Cc*, *Co*, *Ath, Gra* and *Hca*. The x‐ and y‐axes were same as aforementioned in b.

The development of genome sequencing and transcriptome sequencing technologies has provided a great research basis for drawing a sketch of jute bast fibre development regulation (Figure 2a; Table S11). Among the genes we identified and analyzed, thirty genes are related to fibre initiation and elongation, cellulose biosynthesis and lignin biosynthesis. Among the fibre initiation and elongation genes detected in the *C. capsularis* and *C. olitorius* genomes, six genes were highly expressed after gibberellin treatment, and we detected expansions of the *PIN1* and *LBD1* gene families compared with those in the *A. thaliana* genome (Figure 2b). These genes were more highly expressed in stem bark than in leaves (Figure 2b).

The majority of the secondary cell wall in jute fibre is comprised of cellulose, and thus the *cellulose synthase* (*CesA*) genes are one of the most widely used genes for studying cellulose biosynthesis. We identified a total of 10 *CesA* and 26 *cellulose synthase‐like* (*CSL*) genes in the *C. capsularis* and *C. olitorius* genomes, respectively (Figure S9). The expression of the secondary cell wall synthesis‐specific gene *CesA4* was more distinctly elevated in stem bark than in leaves, which together with the expansion of this gene family in jute compared with that in *A. thaliana*, indicates the importance of *CesA4* (*Cc.01G0010420*) for secondary cell wall cellulose biosynthesis in jute (Figure 2c). In contrast, the significantly elevated expression of *CesA3* (*Cc.05G0012040*) in leaves suggested that this gene might be involved in cellulose biosynthesis in the primary cell wall (Figure 2c).

Cellulose synthase‐like (*CSL*) genes, which are highly homologous with *CesA* genes, are also involved in cellulose biosynthesis. The CSL protein‐encoding genes can be divided into six subgroups: *CSLA*, *CSLB*, *CSLC*, *CSLD*, *CSLE* and *CSLG*, and the numbers of homologous genes differ from those found in *A. thaliana*. After subjecting plants to gibberellin treatment, the expression of *CSL* homologous genes in jute stem bark became differentiated. Compared with their transcript abundances in leaves, *CSLA* and *CSLC* were highly expressed in stem bark, *CSLE* and *CSLG* were highly expressed in leaves, and *CSLB* and *CSLD* were both expressed in leaves and stem bark (Figure 2c). These results reflected that *CSL* genes may have undergone some functional differentiation in jute (Figure 2c).

Like genes involved in cellulose biosynthesis, the numbers of homologous genes involved in lignin biosynthesis in jute also differed from those in *A. thaliana*, and we found expansions of the *C4H*, *F5H* and *CCoAOMT* gene families compared with those in the *A. thaliana* genome (Figure 2c). The expression of most lignin biosynthetic genes was significantly decreased after exogenous gibberellin treatment, suggesting that decreased expression of lignin genes might improve the fibre quality of jute (Figure 2c). However, no obvious expansion occurred in the total number of orthologous genes associated with fibre formation in jute that compared with other fibre crops (*G. raimondii* and *H. cannabinus*) (Figure 2b, c).

### Resequencing and discovery of genomic variation

**Figure 3:**
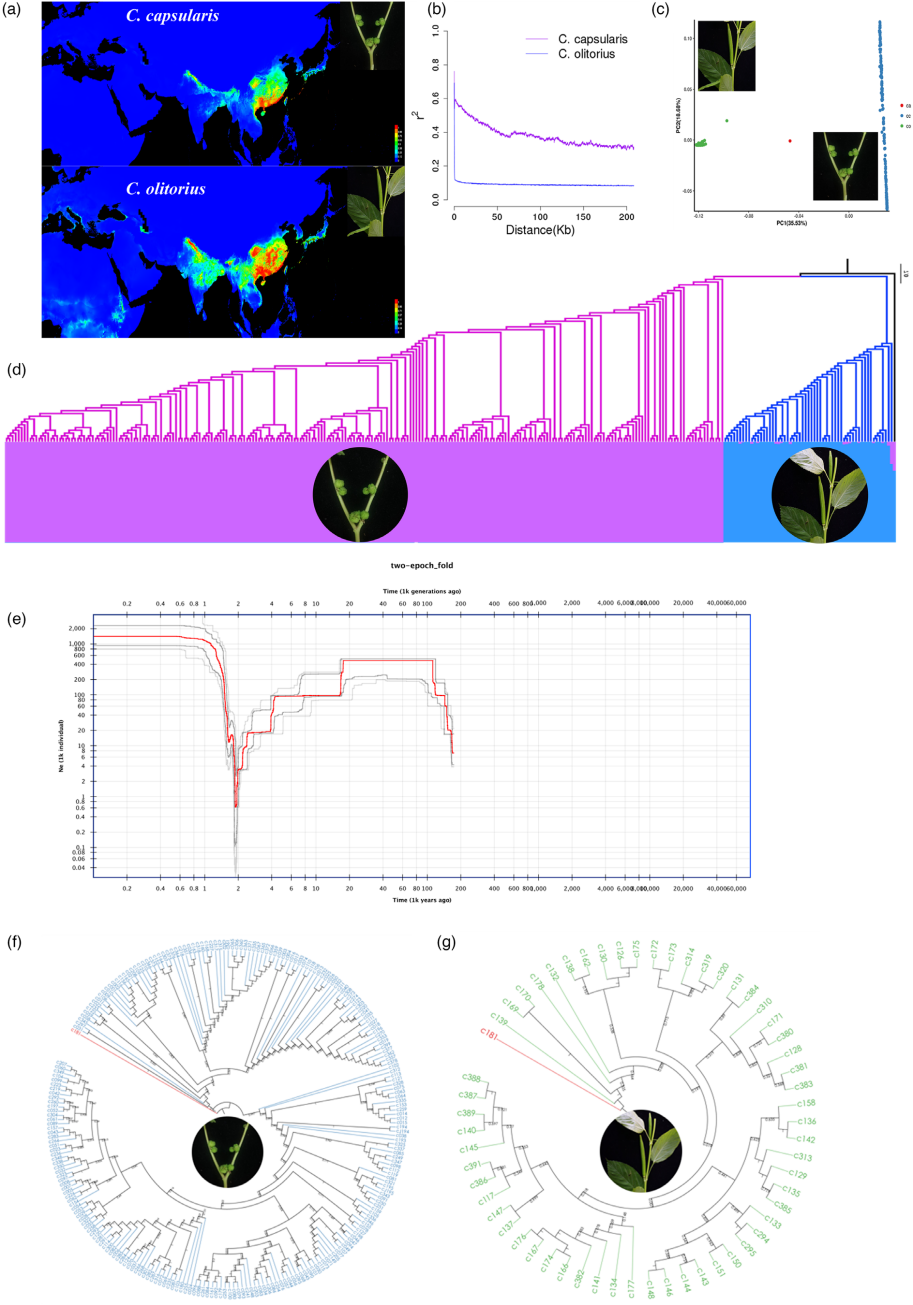
Geographical distribution and population structure of *Corchorus capsularis* and *C. olitorius* populations. (a) The predicted distribution map of *C. capsularis* and *C. olitorius* inferred using their longitude and latitude positions. (b) Linkage disequilibrium (LD) decay revealed different patterns of LD decay for these two species, with values reaching half of the *r^2^* values at 25 and 81 kb for *C. capsularis* and *C. olitorius*, respectively. (c) PCA shows clear separation among the *C. capsularis* and *C. olitorius* populations and *C. aestuans* outgroup. (d) Population admixture showing *K* = 2 using 299 accessions of both *C. capsularis* and *C. olitorius*. (e) Historical effective population size (Ne) for domesticated jute population beginning from 101 KYA (kilo‐years ago) to present. The plot showing jute population has undergone domesticated bottlenecks at ∼2000 years ago. The estimate is median (red line) from 200 bootstrap replicates with 87.5% and 97.5% confidence intervals (four grey lines). (f) Phylogentic relatonships among 242 accessions within *C. capsularis*. (g) Phylogentic relatonships among 57 accessions within *C. olitorius*.

To dissect the genomic basis of fibre quality in jute, we resequenced 300 diverse jute accessions from around the world (with one wild *Corchorus aestuans*, 242 cultivated *C. capsularis* and 57 cultivated *C. olitorius* accessions). From these re‐sequenced genomes, we identified with high confidence 4 113 539 sequence variants that include 3,415,772 single‐nucleotide polymorphisms (SNPs), 344 345 insertions and 353 422 deletions (total 697 767 InDels), for an average of 12.5 variants/kb (Figure 3a; Table S12). We identified 1,259,092 of these variants (15.08%) in intergenic regions, 515 650 synonymous (6.176%) substitutions, 403 392 missense mutations, 4555 nonsense mutations, and 516 169 silent variants. Analysis of linkage disequilibrium (LD) decay shows delayed patterns for two species (Figure 3b), with values reaching to the half of *r* ^2^ values at 24.933 kb and 81 bp for *C. capsularis* and *C. olitorius*, respectively. Population genomic analysis shows a population bottleneck in the genus *Corchorus* that began ∼20 000 years ago with an effective population size (*Ne*) reaching its minimum value ~2000 years ago. After that, the effective population size increased steadily and reached a stable level of about 600 years ago (Figure 3e). Compared with rice, the times at which jute population bottlenecks occurred were close to those for rice, reflecting a concurrence of technological innovations for meeting basic human needs for food and clothing around the world. However, the period of time over which the effective population size of jute continued to decrease and lasted longer than that for rice (Meyer *et al*., 2016).

By setting the wild jute species *C. aestuans* as the outgroup, the phylogenetic relationship among 300 accessions shows that jute accessions can be classified into two divergent groups corresponding to the two cultivated species *C. capsularis* and *C. olitorius*. This classification is supported by the evidence from population PCA (Figure 3c) and admixture analysis (Figure 3d). The PCA (Figure 3c) among jute species clearly separate *C. capsularis and C. olitorius* from their wild congener. Our analysis of population admixture shows the optimal population stratification for these 300 jute accessions to be *K* = 2 or *K* = 3, which clearly separates *C. capsularis* from *C. olitorius* and *C. aestuans* (Figure 3d). Analysis of admixture within each species indicates that the *C. capsularis* population can be divided into 13 subgroups or clusters (Figure S10), while that of *C. olitorius* can be divided into three subgroups or clusters (Figure S10) according to the optimal K values. This conclusion was also supported by the phylogenetic analyses of 242 cultivated *C. capsularis* as well as of 57 cultivated *C. olitorius* accessions using the wild *C. aestuans* as the outgroup for each separately (Figures 3f,g). Our analysis of migration events showed that there was no gene flow between *C. capsularis* and *C. olitorius* after their divergence (Figure S11), which might account for the hybrid incompatibility between *C. capsularis* and *C. olitorius*. However, partial gene flow has occurred within *C. capsularis* (between subgroups *Cc2* and *Cc3*) (Figure S11).

Currently, *C. capsularis* is mainly distributed in tropical Asia including southern China, while *C. olitorius* is mainly distributed in Asia and Africa (Figure 3a). Around the Last Glacial Maximum (LGM), both *C. capsularis* and *C. olitorius* were present in southern Asia, but only *C. olitorius* was present in Africa (Figure S12). The distribution of *C. olitorius* in Africa then began to decrease, while the distribution of jute in Asia, especially in China, has increased because the mid‐Holocene epoch ~2000 years ago (Figure S13). At present, *C. olitorius* has become relatively scarce in Africa, while Asia has become a major producer of jute (Figure 3a). The main reasons for these changes in distributions are rising temperatures and droughts on the African continent, in contrast to abundant rain and more suitable environment for growing jute on the Asian continent. Thus, the changing distribution of jute has been closely related to human activities and global climate changes.

### Selection signals in C. capsularis and C. olitorius

**Figure 4:**
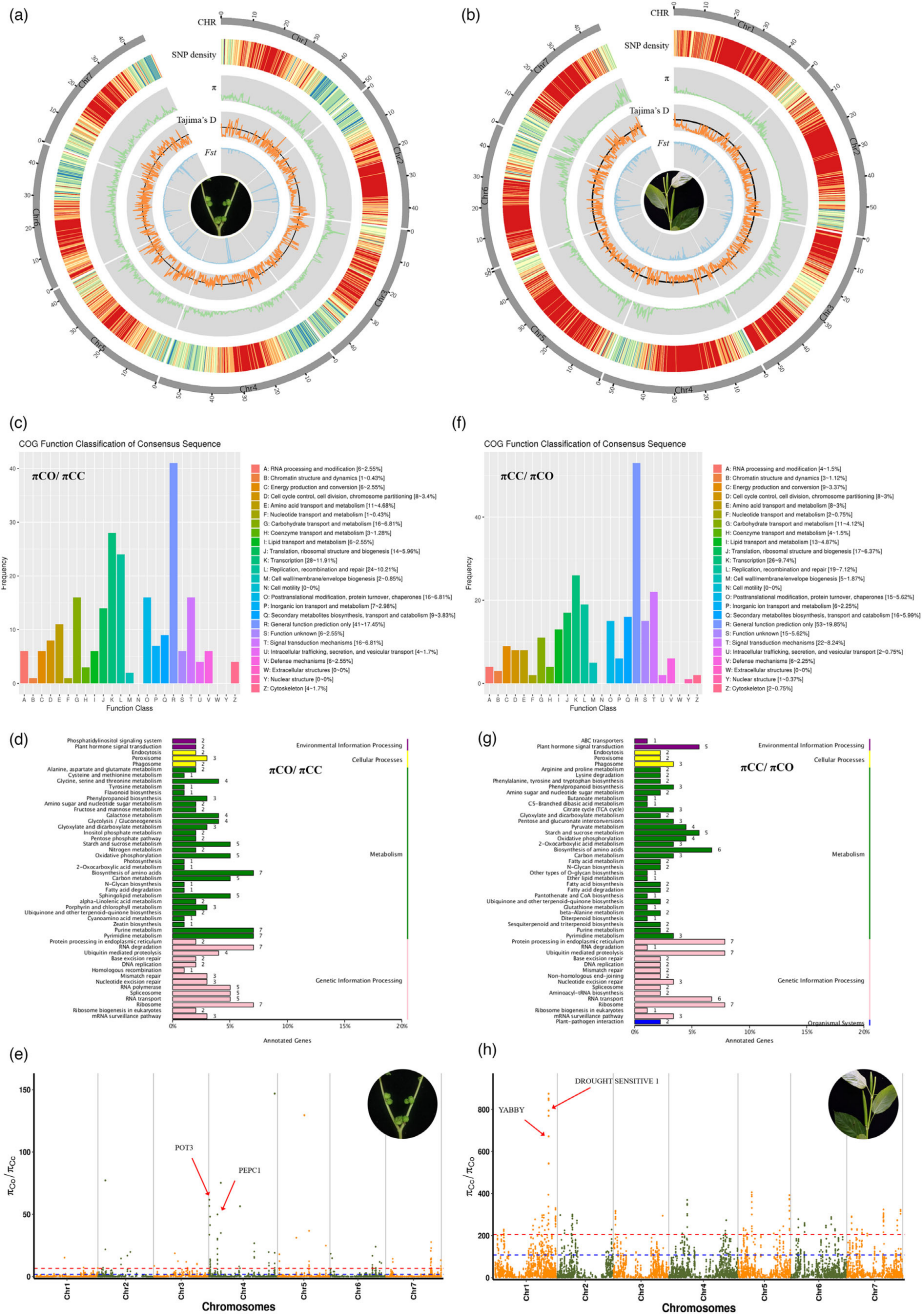
Genome‐wide genetic diversity and divergent selection between *Corchorus capsularis* and *C. olitorius* populations. (a) Population divergence (*F*st) between *C. capsularis* and *C. olitorius* based on *C. capsularis* genome is shown in inner cycle on circos plot. SNP density, nucleotide diversity (π), Tajima’s D of *C. capsularis* populations are shown from outer to center on circos plot. (b) Population divergence (*F*st) between *C. capsularis* and *C. olitorius* based on *C. olitorius* genome is shown in inner cycle on circos plot. SNP density, nucleotide diversity (π), Tajima’s *D* of *C. olitorius* populations are shown from outer to center on circos plot. (c) COG functioal classification of fixed sites under selection in *C. capsularis* population comparing to the *C. olitorius* population. (d) KEGG enrichments and of fixed sites under selection in *C. capsularis* population comparing to the *C. olitorius* population. (e) Manhattan plot of fixed sites under selection in *C. capsularis* population comparing to the *C. olitorius* population. (f) COG functioal classification of fixed sites under selection in *C. olitorius* population comparing to the *C. capsularis* population. (g) KEGG enrichments and fixed sites under selection in *C. olitorius* population comparing to the *C. capsularis* population. (h) Manhattan plot of fixed sites under selection in the *C. olitorius* population comparing to the *C. capsularis* population.

The average SNP density in *C. capsularis* and *C. olitorius* is estimated to be 12.06 and 5.69 kb, respectively (Figure 4a,b). We have estimated the average nucleotide diversities (π) of *C. capsularis* and *C. olitorius* as 0.155 × 10^−3^ and 0.146 × 10^−3^, respectively, suggesting low genetic diversity within the two cultivated species (Figure 4a,b). The average Tajima’s D (Danecek *et al*., 2011) value for *C. capsularis* (−0.796) was higher than that for *C. olitorius* at −1.298 (Figure 4a,b). The average genetic divergence (*F*st) between the two species is 0.970 and 0.963 using the genomes of *C. capsularis* or *C. olitorius* as the reference, respectively (Figure 4a,b). The negative Tajima’s D value in both *C. capsularis* and *C. olitorius* indicates an excess of rare alleles in the population, implying a recent strong selective sweep or population expansion after a recent domestication bottleneck.

In terms of genome‐wide divergent selection between *C. capsularis* and *C. olitorius*, we detected selective signals in *C. capsularis* by comparing the ratios of nucleotide diversity (π_co_/π_cc_) (Danecek *et al*., 2011) between *C. olitorius* and *C. capsularis* populations in 50‐kb sliding windows with a step size of 10 kb, and vice versa for selection signal in *C. olitorius* using π_cc_/π_co_. A total of 513 fixed sites were detected in the *C. capsularis* population and 532 fixed sites were detected in the *C. olitorius* population (Tables S13 and S14). COG functional classification and KEGG enrichment analyses showed that genes with selective signals in *C. capsularis* were enriched mainly for general functions including ‘transcription’, ‘replication’, ‘recombination and repair’, ‘ribosome’, ‘pyrimidine metabolism’, ‘purine metabolism’, biosynthesis of amino acids’, and ‘RNA degradation’ (Figure 4c,d). Among these enriched genes, we identified selective signals in *C. capsularis* for *Cc.01G0000430* (*POT3*: K^+^ uptake transporter 3) and *Cc.01G0000500* (*PEPC1*: Pyridoxal phosphate phosphatase‐related protein) (Figure 4e). The main function of *PEPC1* is to participate in the essential metabolism of amino acids in organisms. *POT3* plays an important role in response salt stress in *C. capsularis*.

However, the genes with selective signals in *C. olitorius* enriched for general functions were predicted to be involved in ‘transcription’, ‘signal transduction mechanisms’, ‘protein processing in the endoplasmic reticulum’, ’ubiquitin mediated proteolysis’, and ’ribosome’ by COG functional classification and KEGG enrichment analyses (Figure 4f,g). The genes with selective signals in *C. olitorius* in *DROUGHT SENSITIVE 1* (homologous to *Cc.01G0033270*) and *YABBY* (homologous to *Cc.01G0033370*) play important roles in the regulation of drought stress and floral organ development, respectively (Figure 4h).

### GWAS identification of loci involved in fibre quality of bast fibre

To identify loci or candidate genes underlying fibre quality, the best linear unbiased prediction (BLUP) for each investigated trait of each accession was obtained using an R script, based on a linear model and corresponding characteristics with three replicates over three years. The frequency distributions of variation in important traits for these 299 accessions grown in three years are shown in Figures S14–S16. The resulting values were used as phenotypes for the genome‐wide association analysis. For the three agronomic traits related to fibre quality (cellulose content, lignin content and fibre fineness), hundreds of putative significant marker‐trait associations (MTAs) were detected (*P* < 0.05) with at least one MTA identified for each trait (Tables S15–S17).

**Figure 5:**
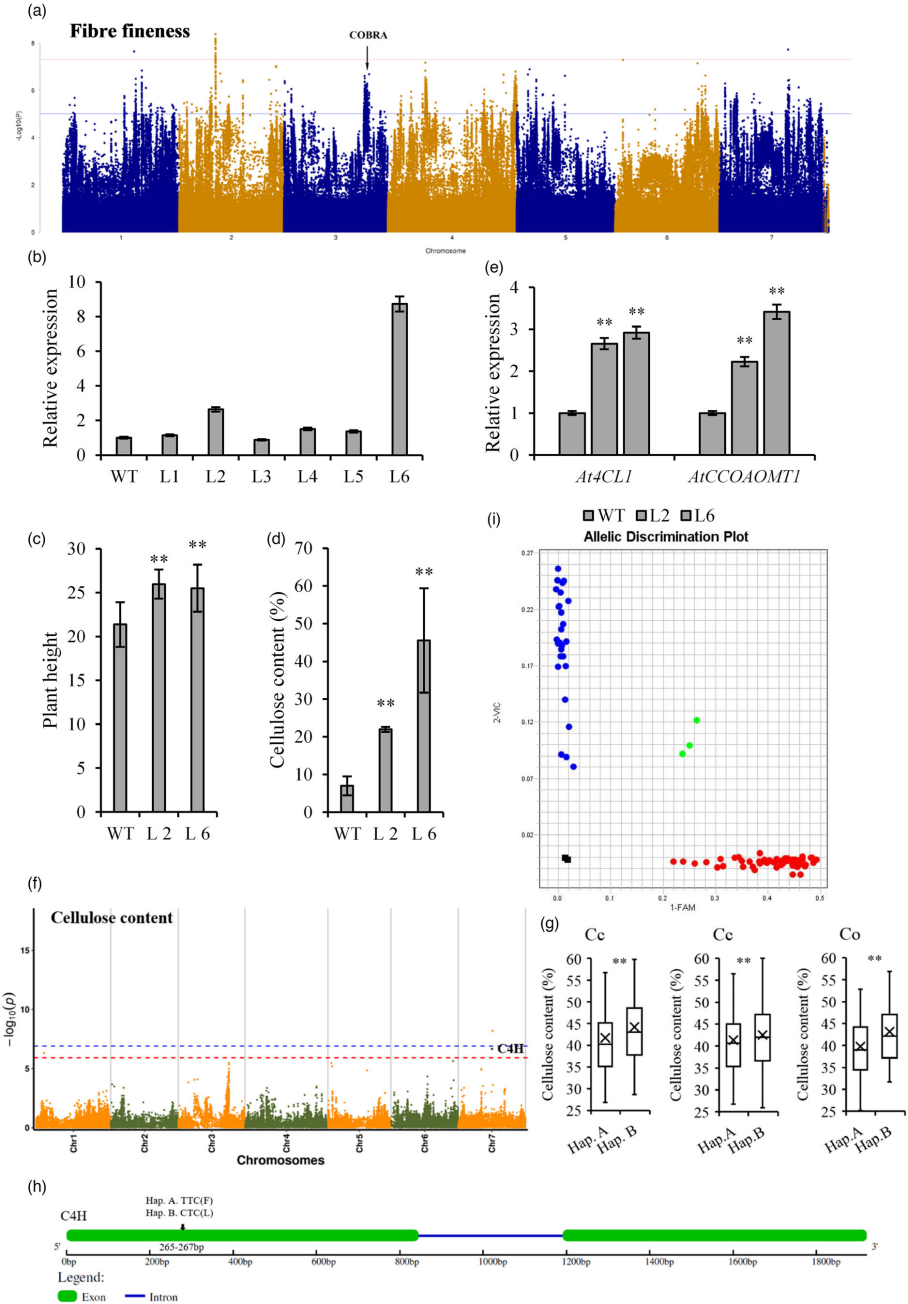
Genome‐wide identification of genes in fibre quality in jute. (a) Manhattan plot showing significant jute locus associated with fibre fineness. Negative log_10_ *^p‐^* ^value^ values from a genome‐wide scan are plotted against position on each of seven chromosomes. Dashed line represents the significance threshold. The COBRA family proteins are known to control the correct location of cellulose microfibrils in cell wall and directional elongation of cells in other plants. (b) The expression analysis of *CcCOBRA1* in transgenic over‐expression lines of *A. thaliana* by qRT‐PCR. (c) The plant height of WT and transgenic over‐expression lines in *A. thaliana*. Error bars, SD (*n* = 3). **P < 0.01(Welch’s *t*‐test). (d) The cellulose content of wild types (WT) and transgenic *A. thaliana* plants. Error bars, SD (*n* = 3). **P < 0.01(Welch’s *t*‐test). (e) The transcription level of lignin marker genes, *At4CL1* and *AtCCOAOMT1,* in transgenic lines and WT plants (data are the means ± SD of three biological replicates). (f) Manhattan plot showing significant jute locus associated with cellulose content of bast fibre. Negative log_10_ *^p‐^* ^value^ values from a genome‐wide scan are plotted against position on each of seven chromosomes. Dashed line represents the significance threshold. (g) Boxplots for cellulose content based on the genotypes of *CcC4H1* in different jute populations. Box edges represent the 0.25 quantile and 0.75 quantile with the median values shown by bold lines. Whiskers extend to data no more than 1.5 times the interquartile range, and remaining data are indicated by dots. Differences between the genotypes were analyzed by Welch’s *t*‐test. **P < 0.01. *Cc*, *C. capsularis*; *Co*, *C. olitorius*. (h) Gene structure and genetic variants in a candidate gene *CcC4H1*. Exons and introns are represented by boxes and lines, respectively. The position of the possibly causal micro‐structure variation is marked. (i) Genotyping of *CcC4H1* gene in jute population by Kompetitive Allele Specific PCR (KASP). Blue and red points represent two homozygous genotype, green points represent parental complementary genotype, black squares represent non‐template control (NTC).

Fibre fineness is an important index of textile fibre and yarn, which have a great influence on fabric style, handling and processing. However, the genetic characterization of this trait has not been thoroughly documented, and only some QTL for fibre fineness have been identified and reported in jute (Das *et al*., 2012; Topdar *et al*., 2013). During GWAS of fibre fineness, we detected a strong signal in chromosome 3 (Figure 5a; Table S15; Figure S17). In particular, among the genes within the strong peak, *Cc.03G0022380* encodes a COBRA family protein, which is known to control the correct location of cellulose microfibrils in cell wall and directional elongation of cells in other plant species. Therefore, the cellulose content will decrease when the *Cobra* gene mutates. We found that the expression level of *CcCOBRA1* (*Cc.03G0022380*) increased with the accumulation of bast fibre in jute (Figure S18), indicating that this gene might represent an important locus controlling fibre quality in jute.

Then, we examined the function of *CcCOBRA1* by constructing the 35S::*CcCOBRA1* plant transformation vector and generating transgenic *A. thaliana* lines (Figure 5b). Compared to wild types (WT), over‐expression of *CcCOBRA1* in the two highest expressing transgenic *A. thaliana* lines (L2 and L6) resulted in a significant increase in plant height (*P* < 0.01) (Figure 5b,c). The cellulose content of selected transgenic lines (L2 and L6) and WT plants was determined at the initial flowering stage. The over‐expression of *CcCOBRA1* resulted in a significant increase in cellulose content in transgenic lines compared with WT (Figure 5d). Furthermore, the transcription level of marker genes related to fibre development (*At4CL1* and *AtCCOAOMT1*) was measured by qRT‐PCR. The transgenic lines displayed a significant increase compared with WT plants, indicating a positive regulation of these two genes by *CcCOBRA1* (Figure 5e). These findings further demonstrate that the *CcCOBRA1* gene is probably involved in facilitating interactions among various members of fibre development related protein families to mediate fibre quality in plants.

Cellulose content and lignin content are other important indicators of jute fibre quality in bast fibre crops. In our study, a significant SNP in cellulose content and lignin content was located in a gene (*Cc.07G0016270*) in chromosome 7, which exhibited a high −log_10_ *^p‐^* ^value^ and encodes a cinnamate‐4‐hydroxylase protein (C4H) (Figure 5f; Tables S16 and S17; Figure S19). C4H that mediates the conversion of cinnamate to p‐coumarate is a key enzyme of the ‘shikimate and aromatic amino acid biosynthetic pathways’ of the phenylpropanoid biosynthetic pathway, and it is also one of the most widely distributed CYP450 involved in lignin synthesis in plants (Zhang *et al*., 2020a). Moreover, haplotype analysis showed that the haplotype B (CTC, L) was mainly found in accessions with high‐cellulose content, whereas haplotype A (TTC, F) was found in accessions with low‐cellulose content (Figure 5g,h). In addition, we found that the expressions of *CcC4H1* in stem bark were significantly higher than that in leaves (Figure S20), indicating that it might be possible to decrease lignin content in jute by down‐regulating *CcC4H1* expression and thereby greatly improve fibre quality.

To further validate the relationship between the point mutation and fibre quality, we performed KASP assays on this SNP. Based on this SNP locus, the KASP marker was used to genotype 299 jute accessions. KASP assay was able to demarcate low‐lignin allele from high‐lignin allele (Figure 5i; Table S18). Moreover, we developed an F_2_‐segregated population derived by crossing two germplasms, namely cultivated Huangma 179 (an elite cultivar) and Aidianyehuangma (a typical dwarf cultivar). In this population, KASP assays further validated the correlation of fibre quality with the single C‐to‐T mutation in the exon of *CcC4H1*. Such a complete association between the SNP in *CcC4H1* and the fibre quality phenotype implies that this SNP is possibly the causal mutation for the fibre quality difference in jute. Therefore, the manipulation of genes associated with fibre quality identified by GWAS should have practical applications in jute breeding for high fibre quality.

### Selective sweeps under domestication

**Figure 6:**
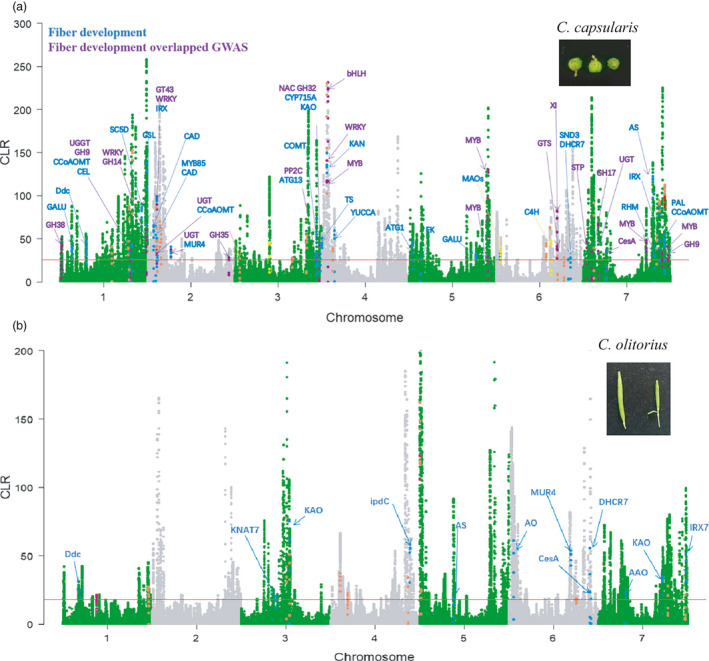
Selective swept genes related to fibre quality traits in *C. capsularis* and *C. olitorius* populations. (a) Genome‐wide selective sweeps in cultivated *C. capsularis* showing 5% cutoff outlier of absolute CLR statistics across reference genomes, respectively. (b) Genome‐wide selective sweeps in cultivated *C. olitorius* showing 5% cutoff outlier of absolute CLR statistics across reference genomes, respectively. The red solid line indicates the candidate regions above the 5% cutoff outlier with significant deviations from neutrality. The highlighted signals showing overlapped genes identified in controlling the fibre development using gene family analysis and GWAS mapping.

To detect selective sweeps that occurred during jute domestication involved in fibre development, we scanned for genomic‐wide selective sweep patterns in both *C. capsularis* and *C. olitorius* using CLR (composite likelihood ratio) statistics (Figure 6). The reference genomes for both *C. capsularis* and *C. olitorius* germplasms were scanned in 20‐kb sliding windows on each chromosome, and the top 5% CLR outlier regions were designated as potential selective sweeps. A total of 2527 genes in *C. capsularis* and 1475 genes in *C. olitorius* populations were identified as having undergone selection (Tables S19 and S20). In the species *C. capsularis*, genes that underwent a selective sweep were significantly enriched for GO terms related to ‘cell cortex’, ‘cell division’, ‘regulation of meristem growth’, ‘peptidase activity’, ‘nitrogen compound metabolic process’, and ‘regulation of meristem growth’ according to our GO term enrichment analysis (*P* < 0.05, Fisher’s exact test) (Table S21; Figure S21). However, in the species *C. olitorius*, these genes that underwent a selective sweep were significantly enriched in for GO terms related to ‘regulation of cell morphogenesis’, ‘ion transport’, ‘pollination’, ‘oxidoreductase activity’, ‘symporter activity’, and ‘catalytic activity’ according to our GO term enrichment analysis (*P* < 0.05, Fisher’s exact test) (Table S22; Figure S22). The genes that experienced selective sweeps in jute are annotated with functions in fibre development and growth.

Among the genes that had undergone selective sweeps in *C. capsularis*, 62 were annotated in pathways related to cellulose synthesis and fibre development (Figure 6a). These genes included two *CesA* (cellulose synthase), two *CSL* (cellulose synthase‐like), two *PE* (pectinesterase) genes, as well as five *MYB* transcription factors that have already been verified to be involved in the SCW regulatory networks in jute. Our GWAS analysis revealed 26 genes that overlap with regions mapped in our GWAS related to fibre quality. This GWAS mapped transcriptional factors (five *MYB*, three *WRKY* and one *bHLH*) that showed strong selective signals suggesting strong artificial selection during domestication. However, in *C. olitorius*, selective sweeps affected 12 genes that likely participate in cellulose synthesis and fibre development pathways (Figure 6b).

## Discussion

Because genetic studies of jute had so far been limited by relatively few genomic resources, we generated approximately 41 Gb (~120× coverage) and 41 Gb (~112× coverage) of raw single‐molecule real‐time (SMRT) data for the two species *Corchorus capsularis* and *C. olitorius* using PacBio Sequel in this study. We then used Hi‐C to generate improved high‐quality chromosome‐level genome assemblies of *C. capsularis* var. HM179 and *C. olitorius* var. KYCG for this study. The new jute reference genomes have 33‐fold higher contiguity than the previously published draft genome assembly of jute (Islam *et al*., 2017), with contig N50 values of 3.2 Mb in the current assembly versus 0.046 Mb in the previous assembly for *C. capsularis*, and 1.5 Mb versus 0.045 Mb in *C. olitorius*). Moreover, the new genome assembly represents a 43‐fold increase in mean contig lengths compared to that in the previous study (Islam *et al*., 2017). Our reference assembly also vastly improved the coverage of regulatory sequences, decreasing the number of genes exhibiting gaps. Moreover, our reference assembly facilitate to identify large structural variants by a direct comparative genome analysis of the two accessions. Interestingly, we identified 13 putative inversions that were larger than 1 Mb, which might partly explain the difference of phenotypic variations between *Corchorus capsularis* and *C. olitorius*. Many previously identified inversion variants in genome have already been linked to phenotypic variations (Feuk *et al*., 2005). Further studies designed to understand the impacts of the inversions identified in this study could allow better understanding of gene expression located in these inversion variants and facilitate investigation on differences of phenotypic variants between the two cultivated jute species. Totally, this improved genomic information for jute will provide a useful resource for exploring important genes and improving our understanding of their evolutionary history. It will also help us to understand the mechanisms that control gene expression of various traits and allow us to identify as well as predict functional genetic variation (Chia *et al*., 2012; Dooner and He, 2008; Zhang *et al*., 2020b).

Identification of candidate genes involved in fibre development or quality is very important for jute genetic improvement. In this study, RNA‐seq showed that the numbers of most fibre‐related genes identified in cultivated jute were comparable to those in *Arabidopsis* and other fibre crops. This suggests that these gene families did not undergo expansion in jute. Therefore, the quality of fibre produced in jute might be related to regulation of gene expression rather than the numbers of genes involved in fibre synthesis.

Further, we also identified hundreds of putative significant marker‐trait associations (MTAs) and candidate genes in jute that are likely related to fibre quality by GWAS. Primary function analysis of two of them, *CcC4H1* and *CcCOBRA1*, were conducted. *CcC4H1* is an important hydroxylase in lignin biosynthesis (Zhang *et al*., 2020a) and high lignin content can negatively affect the quality of bast fibre (Meshram and Palit, 2013). Mutations in *BC1* (encodes a COBRA‐like protein in rice) cause not only a reduction in cell wall thickness and cellulose content but also an increase in lignin level, suggesting that *BC1*, a gene that controls the mechanical strength, plays an important role in the biosynthesis of the cell walls of mechanical tissues (Li *et al*., 2003). Therefore, silencing the *CcC4H1* and overexpression the *CcCOBRA1* might effectively improve the quality of bast fibre in jute.

At the same time, with the aid of the genomic assemblies of two jute subpopulations (*C. capsularis* and *C. olitorius*) and a wild species (*C. aestuans*), we identified regions containing several genes that could have important functions in adaptation and growth in jute (Swarts *et al*., 2017). Among these genes, 26 overlapped with regions related to fibre development‐related traits genes mapped by GWAS. The GWAS mapped regions contain genes encoding transcription factors including five *MYB*, three *WRKY* and one *bHLH* showing strong selective signals that might have undergone strong artificial selection during domestication (Zhou *et al*., 2017). Previous studies revealed that the transcription factor families, notably *MYB* and *WRKY*, play significant roles in various plant biological processes. For example, the *MYB46/MYB83* could activate secondary cell wall biosynthesis in fibres (Guo *et al*., 2017). ABA‐induced WRKY genes can synergistically interact to control GAMYB‐intermediated gibberellin (GA) signaling, and thus creating a novel mechanism for abscisic acid and gibberellin signaling (Xie *et al*., 2006). Although a larger number of jute populations were used in this study compared to previous studies (Sarkar *et al*., 2019), to identify the key genes involved in jute domestication, analyses of more wild jute accessions and related species will be required in the near future.

The high‐quality chromosomal‐level genome sequence assemblies presented here, coupled with access to jute plant materials (242 *C. capsularis* and 57 *C. olitorius* varieties) and experimental fields for conducting experiments in China, allow us to provide valuable resources for understanding the origin and improvement history of jute. These resources will facilitate the dissection of the genetic bases of important and complex agronomic traits in jute including fibre quality. The significant SNPs associated with selection signals and candidate genes that we identified here will be a valuable resource for the improvement of jute and other allied fibre crops.

## Experimental procedures

Materials and Methods as well as any associated references are available in the online version of the paper. The whole‐genome sequence data reported in this paper have been deposited in the Genome Warehouse in National Genomics Data Center, Beijing Institute of Genomics, Chinese Academy of Sciences/China National Center for Bioinformation, under accession number GWHBCLB00000000 and GWHBCLC00000000 that are publicly accessible at https://bigd.big.ac.cn/gwh. All raw sequence data of RNA‐seq are accessible through the NCBI Sequence Read Archive (SRA) under accession PRJNA555734.

## Conflict of interest

The authors declare no competing financial interests.

## Author contributions

Liwu Z., J.Q. and R.M. jointly supervised the work. X.Z., J.Y. and S.C. performed sequencing, assembly and genome annotation. X.Z., S.C. and X.M. performed genome analysis. Lilan Z., Y.X., J.Y. and A.K.I. prepared DNA and RNA samples and performed PCR analysis. J.Q., J.X., A.T., P.F. and L.L. provided the homozygous seeds. Liemei Z., Y.C. and Q.Z. performed transcriptome and gene functional analyses. X.M., H.H., Y.L., X.X., J.W. and Y.X. performed the population genomics, selection analysis and GO enrichments. S.N., S.C., X.Y., Q.H. and Lilan Z. performed phenotyping and contributed to data analysis. Liwu Z. conceived the project. Lilan Z. wrote the manuscript. Liwu Z. and R.M. revised the manuscript. All authors contributed to the revision of the manuscript.

## Supporting information


**Figure S1** Features of *C. capsularis* var. ‘Huangma 179’ (HM179) and *C. olitorius* var. ‘Kuanyechangguo’ (KYCG).
**Figure S2** Estimation of *C. capsularis* and *C. olitorius* genome size based on 19 K‐mer analysis.
**Figure S3** Hi‐C heatmap of *C. capsularis (Cc*) and *C. olitorius* (*Co*) using 150 kb resolution.
**Figure S4** Estimation of the LTR burst time based on intact LTRs identified by LTR_retriever.
**Figure S5** The scatter diagram of Ka (non‐synonymous) and Ks (synonymous) nucleotide substitutions among *C. capsularis* (*Cc*) and *C. olitorius* (*Co*) as well as *Gossypium raimondii* (*Gr*).
**Figure S6** Synthenic analysis between *C. capsularis* (*Cc*) and *C. olitorius* (*Co*).
**Figure S7** GO pathway enrichment analysis of genes located in the inversions between *C. capsularis* and *C. olitorius*.
**Figure S8** Basic GO enrichment information of genes located in the inversions.
**Figure S9** The phylogeny of cellulose synthase (*CesA*) and cellulose synthase‐like (*Csl*) genes in *C. capsularis*, *C. olitorius* and *A. thaliana*.
**Figure S10** Population structure and admixture analysis among 299 accessions in jute.
**Figure S11** The gene exchange in jute natural population during the evolution.
**Figure S12** The predicted distribution map of *C. capsularis* and *C. olitorius* inferred using their longitude and latitude positions in Last Glacial Maximum (LGM).
**Figure S13** The predicted distribution map of *C. capsularis* and *C. olitorius* inferred using their longitude and latitude positions in mid‐Holocene.
**Figure S14** The frequency distribution of cellulose content of bast fibre in 299 jute accessions grown in three years.
**Figure S15** The frequency distribution of lignin content in 299 jute accessions grown in three years.
**Figure S16** The frequency distribution of fibre fineness in 299 jute accessions grown in three years.
**Figure S17** Genome‐wide association studies of fibre fineness in 299 jute accessions grown in different years.
**Figure S18** Relative RNA‐seq expression comparison of *CcCOBRA1*, one GWAS candidate gene, of stem barks at different development stage.
**Figure S19** Genome‐wide association studies of cellulose content in 299 jute accessions grown in different years.
**Figure S20** Relative RNA‐seq expression comparison of *CcC4H1*, one GWAS candidate gene, of leaves and stem barks between *C. capsularis* and *C. olitorius* at the vigorous growth stage (60 days after sowing). Data are means ± SD calculated from three biological replicates (*P*<0.01).
**Figure S21** GO pathway enrichment analysis for the candidate genes in the overlapped improvement and selection outliers detected between different geographical sources in *C. capsularis*.
**Figure S22** GO pathway enrichment analysis for the candidate genes in the overlapped improvement and selection outliers detected between different geographical sources in *C. olitorius*.

**Table S1** Pacbio sequencing of *C. capsularis* and *C. olitorius*.
**Table S2** Statistics of Hi‐C sequencing and mapping of *C. capsularis* and *C. olitorius*.
**Table S3** Statistics of chromosome‐anchored contigs for *C. capsularis* and *C. olitorius*.
**Table S4** BUSCO genome assembly of *C. capsularis* and *C. olitorius*.
**Table S5** BUSCO gene annotation analysis of *C. capsularis* and *C. olitorius*.
**Table S6** Complete genome of *C. capsularis* and *C. olitorius* based on CEGMA.
**Table S7** Statistics of complete gene annotations of *C.capsularis* and *C.olitorius*.
**Table S8** Summary analysis of different types of transposable elements in *C. capsularis* and *C. olitorius* genome.
**Table S9** Phylogenetic analysis of *Malvaceae* family.
**Table S10** The GO enrichments of inversion genes between *C. capsularis* and *C.olitorius*.
**Table S11** Genes involved in bast fibre formation in *Corchorus* sp.
**Table S12** List of 300 jute accessions.
**Table S13** Fixed sites under selection in *C. capsularis* compared to the *C. olitorius* populations.
**Table S14** Fixed sites under selection in *C. olitorius* compared to the *C. capsularis* populations.
**Table S15** Candidate genes of fibre fineness of bast fibre in the genome‐wide association studies.
**Table S16** Candidate genes of cellulose content of bast fibre in the genome‐wide association studies.
**Table S17** Candidate genes of lignin content of bast fibre in the genome‐wide association studies.
**Table S18** Kompetitive Allele Specific PCR (KASP) Primers and sequences.
**Table S19** Candidate genes from the overlapped improvement‐selection outliers detected between different geographical sources in *C. olitorius*.
**Table S20** Candidate genes from the overlapped improvement‐selection outliers detected between different geographical sources in *C. capsularis*.
**Table S21** GO enrichments of candidate genes from the overlapped improvement‐selection outliers detected between different geographical sources in *C. capsularis*.
**Table S22** GO enrichments of candidate genes from the overlapped improvement‐selection outliers detected between different geographical sources in *C. olitorius*.

**Appendix S1** Materials and Methods.
