# Supplementary material for: Reference genomes of the two cultivated jute species
Source: Plant Biotechnol J. 2021 Jul 8;19(11):2235–48. doi: 10.1111/pbi.13652 (PMC8541789; doi:10.1111/pbi.13652)
Supplement: Supplementary file 1 — Figure S1 Features of C. capsularis var. ‘Huangma 179’ (HM179) and C. olitorius var. ‘Kuanyechangguo’ (KYCG). Figure S2 Estimation of C. capsularis and C. olitorius genome size based on 19 K‐mer analysis. Figure S3 Hi‐C heatmap of C. capsularis (Cc) and C. olitorius (Co) using 150 kb resolution. Figure S4 Estimation of the LTR burst time based on intact LTRs identified by LTR_retriever. Figure S5 The scatter diagram of Ka (non‐synonymous) and Ks (synonymous) nucleotide substitutions among C. capsularis (Cc) and C. olitorius (Co) as well as Gossypium raimondii (Gr). Figure S6 Synthenic analysis between C. capsularis (Cc) and C. olitorius (Co). Figure S7 GO pathway enrichment analysis of genes located in the inversions between C. capsularis and C. olitorius. Figure S8 Basic GO enrichment information of genes located in the inversions. Figure S9 The phylogeny of cellulose synthase (CesA) and cellulose synthase‐like (Csl) genes in C. capsularis, C. olitorius and A. thaliana. Figure S10 Population structure and admixture analysis among 299 accessions in jute. Figure S11 The gene exchange in jute natural population during the evolution. Figure S12 The predicted distribution map of C. capsularis and C. olitorius inferred using their longitude and latitude positions in Last Glacial Maximum (LGM). Figure S13 The predicted distribution map of C. capsularis and C. olitorius inferred using their longitude and latitude positions in mid‐Holocene. Figure S14 The frequency distribution of cellulose content of bast fibre in 299 jute accessions grown in three years. Figure S15 The frequency distribution of lignin content in 299 jute accessions grown in three years. Figure S16 The frequency distribution of fibre fineness in 299 jute accessions grown in three years. Figure S17 Genome‐wide association studies of fibre fineness in 299 jute accessions grown in different years. Figure S18 Relative RNA‐seq expression comparison of CcCOBRA1, one GWAS candidate gene, of stem barks at differen [file PBI-19-2235-s002.pptx]

## Slide 1
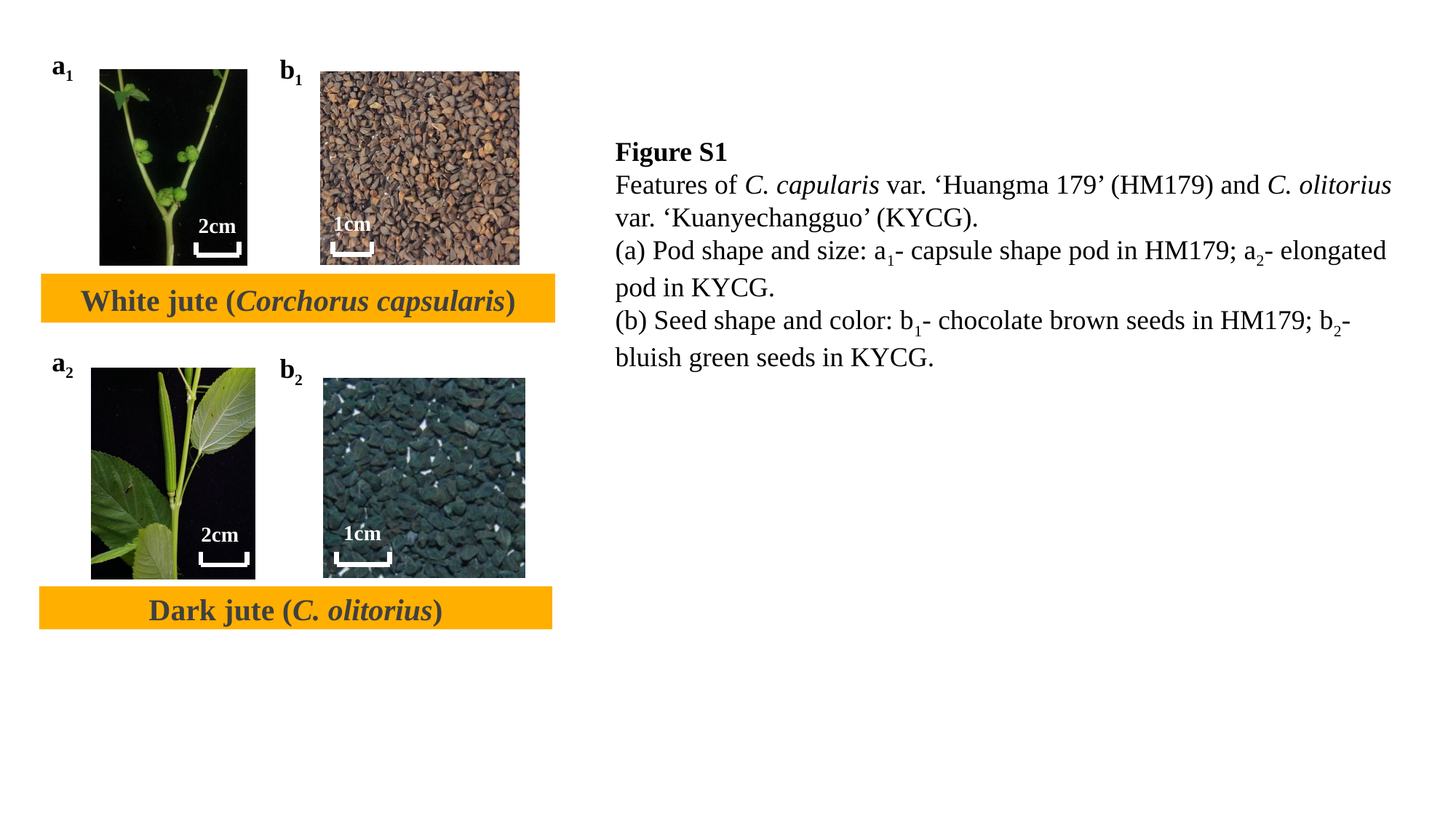

a1
b1
Figure S1
Features of C. capularis var. ‘Huangma 179’ (HM179) and C. olitorius var. ‘Kuanyechangguo’ (KYCG).
(a) Pod shape and size: a1- capsule shape pod in HM179; a2- elongated pod in KYCG.
(b) Seed shape and color: b1- chocolate brown seeds in HM179; b2- bluish green seeds in KYCG.
1cm
2cm
White jute (Corchorus capsularis)
a2
b2
1cm
2cm
Dark jute (C. olitorius)

## Slide 2
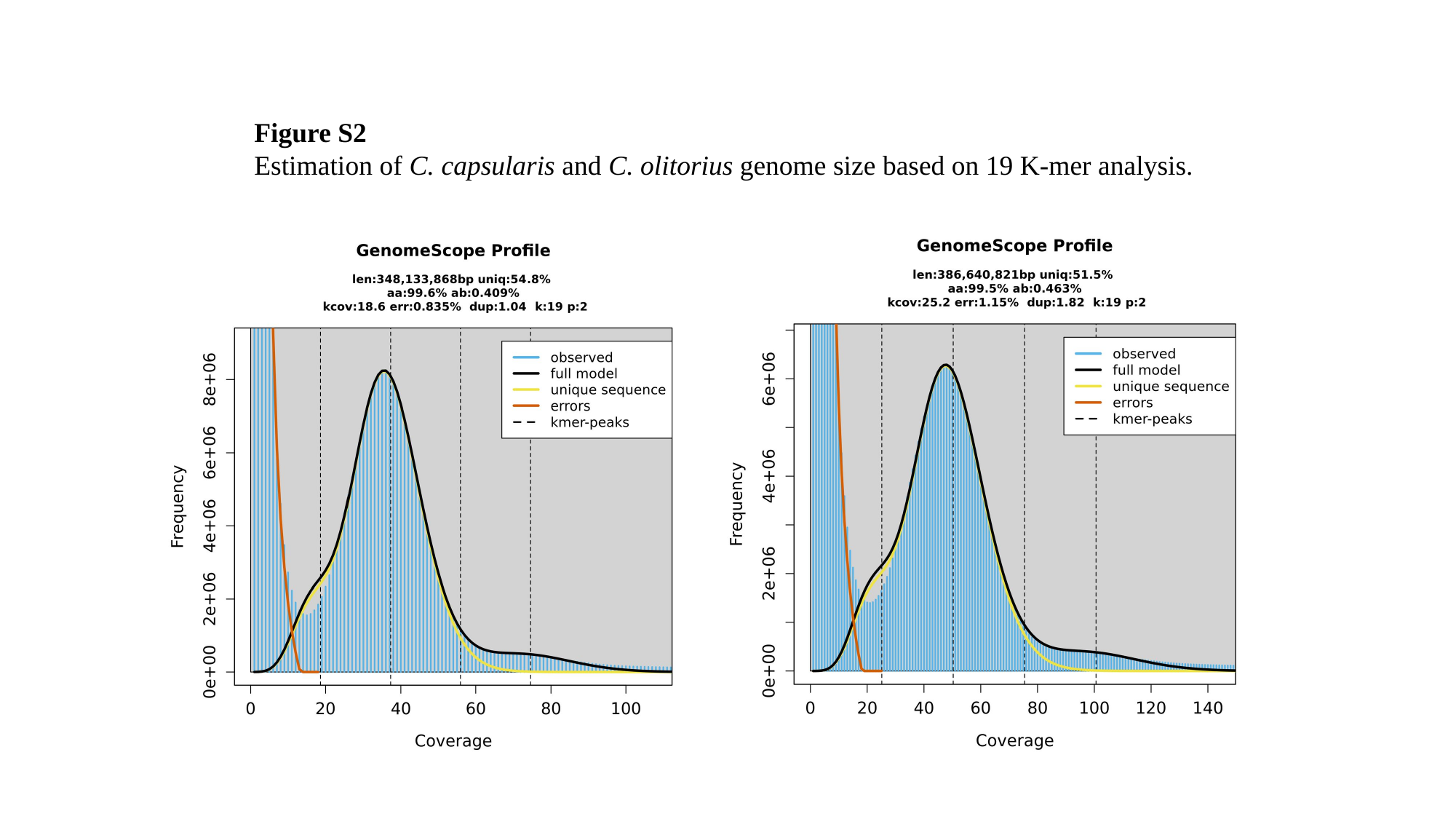

Figure S2
Estimation of C. capsularis and C. olitorius genome size based on 19 K-mer analysis.

## Slide 3
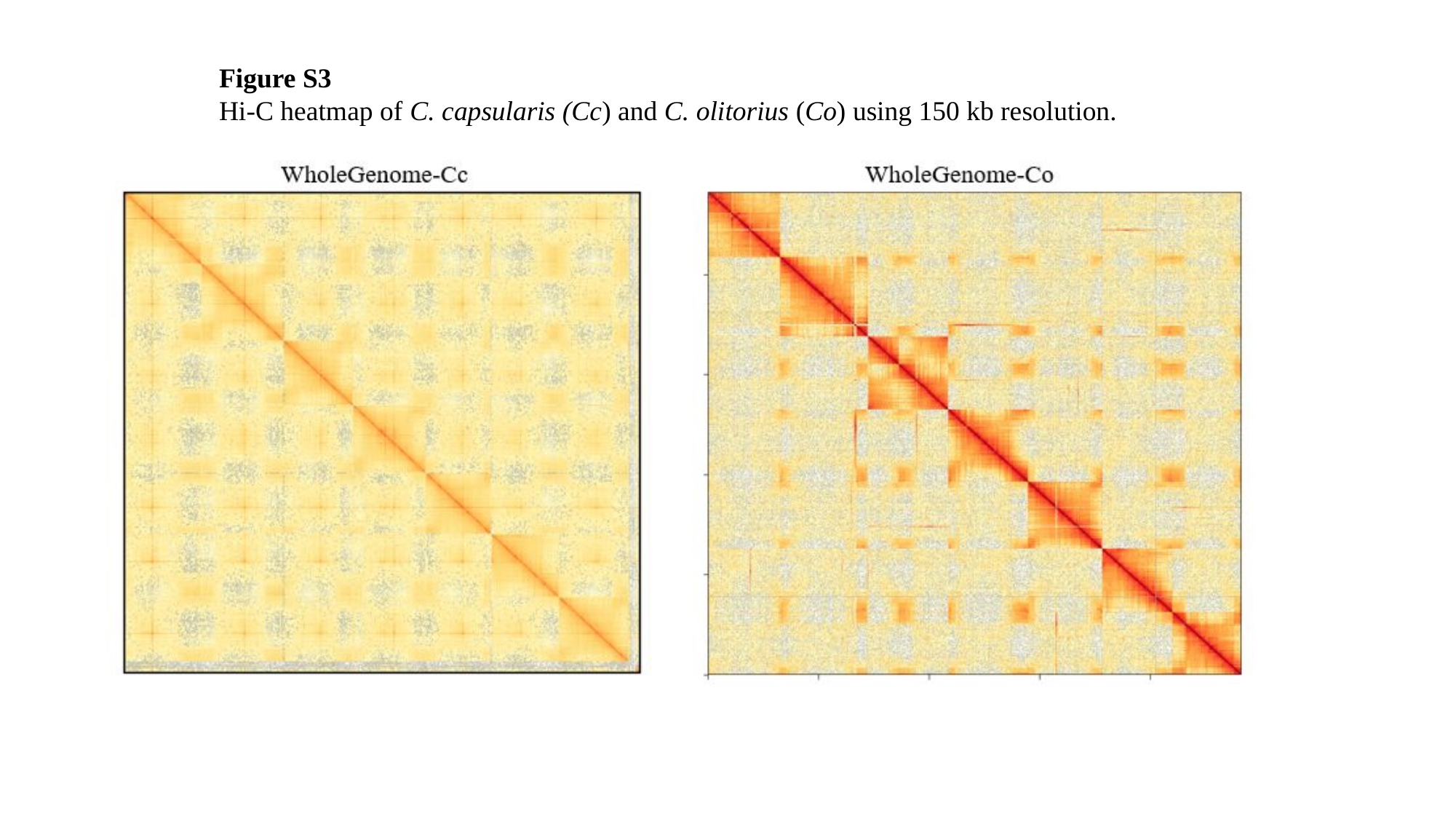

Figure S3
Hi-C heatmap of C. capsularis (Cc) and C. olitorius (Co) using 150 kb resolution.

## Slide 4
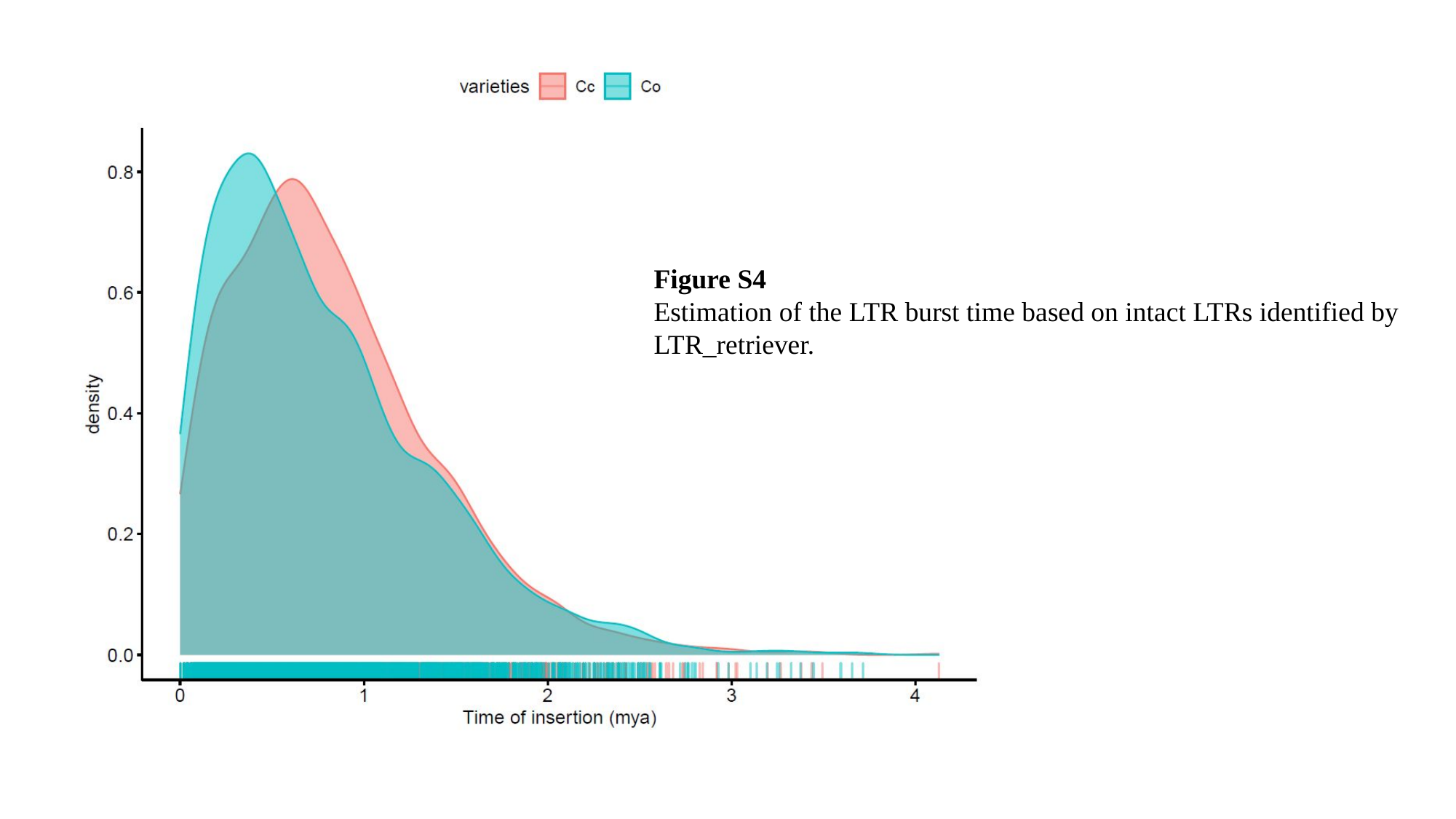

Figure S4
Estimation of the LTR burst time based on intact LTRs identified by LTR_retriever.

## Slide 5
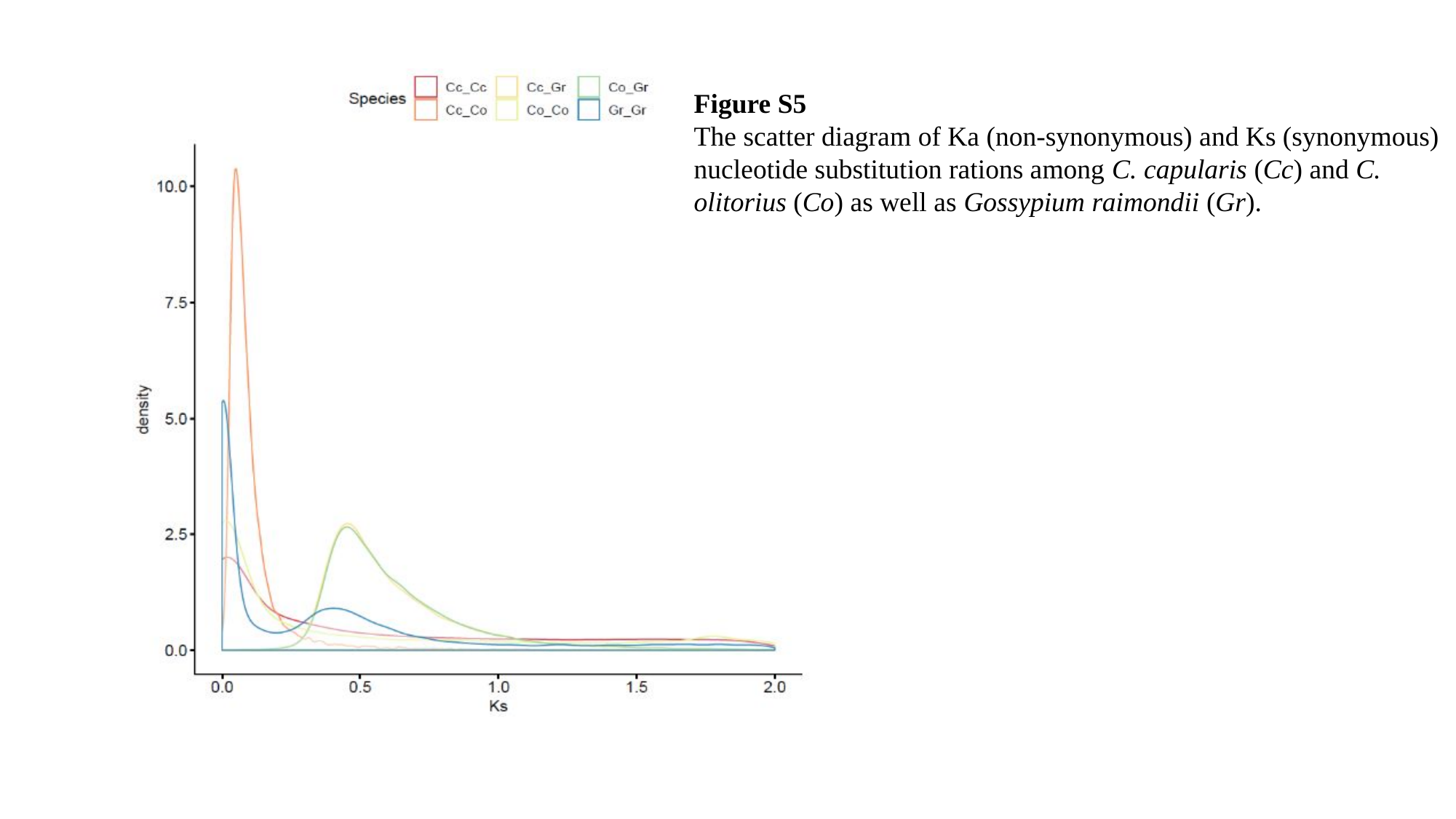

Figure S5
The scatter diagram of Ka (non-synonymous) and Ks (synonymous) nucleotide substitution rations among C. capularis (Cc) and C. olitorius (Co) as well as Gossypium raimondii (Gr).

## Slide 6
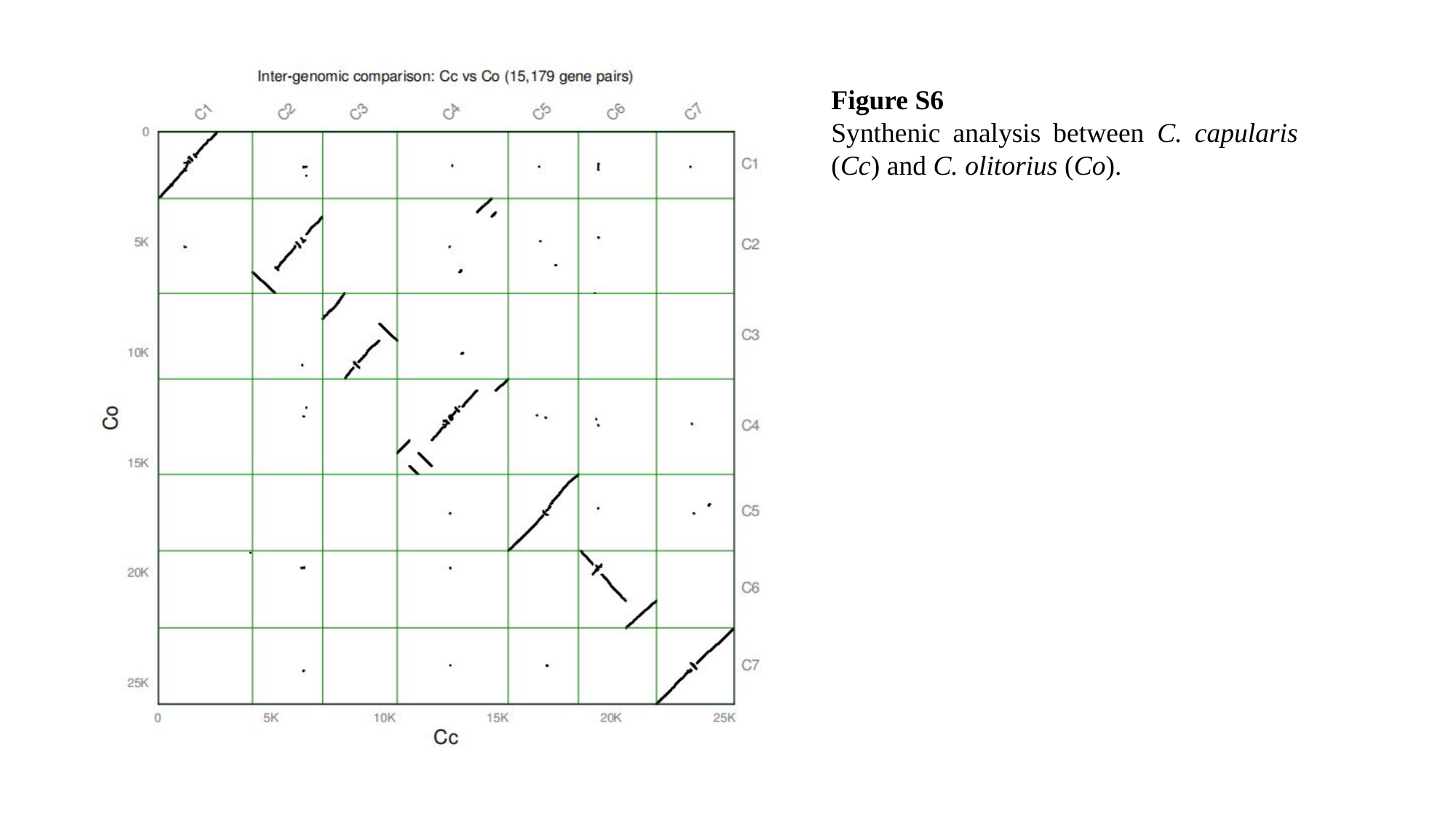

Figure S6
Synthenic analysis between C. capularis (Cc) and C. olitorius (Co).

## Slide 7
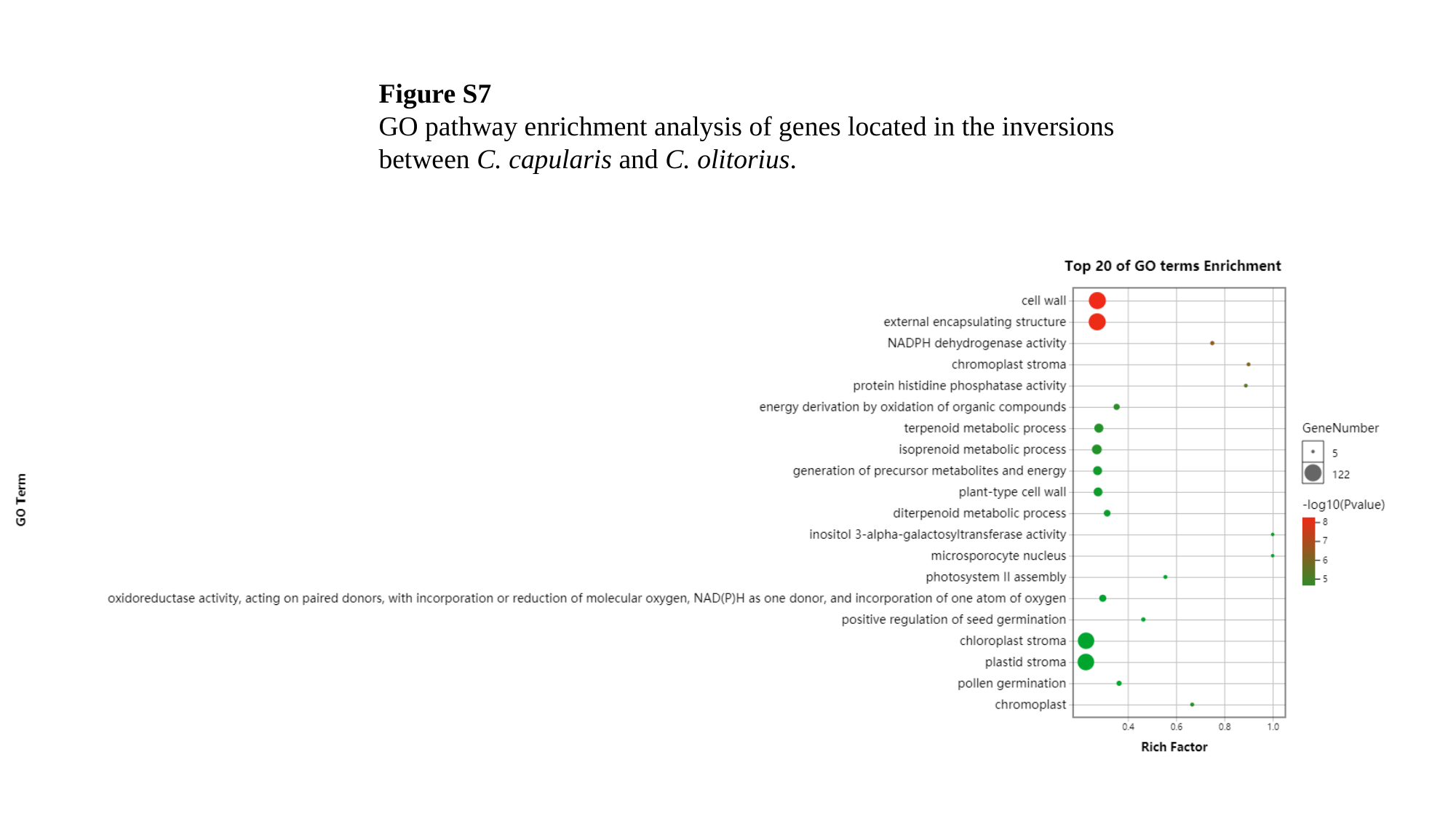

Figure S7
GO pathway enrichment analysis of genes located in the inversions between C. capularis and C. olitorius.

## Slide 8
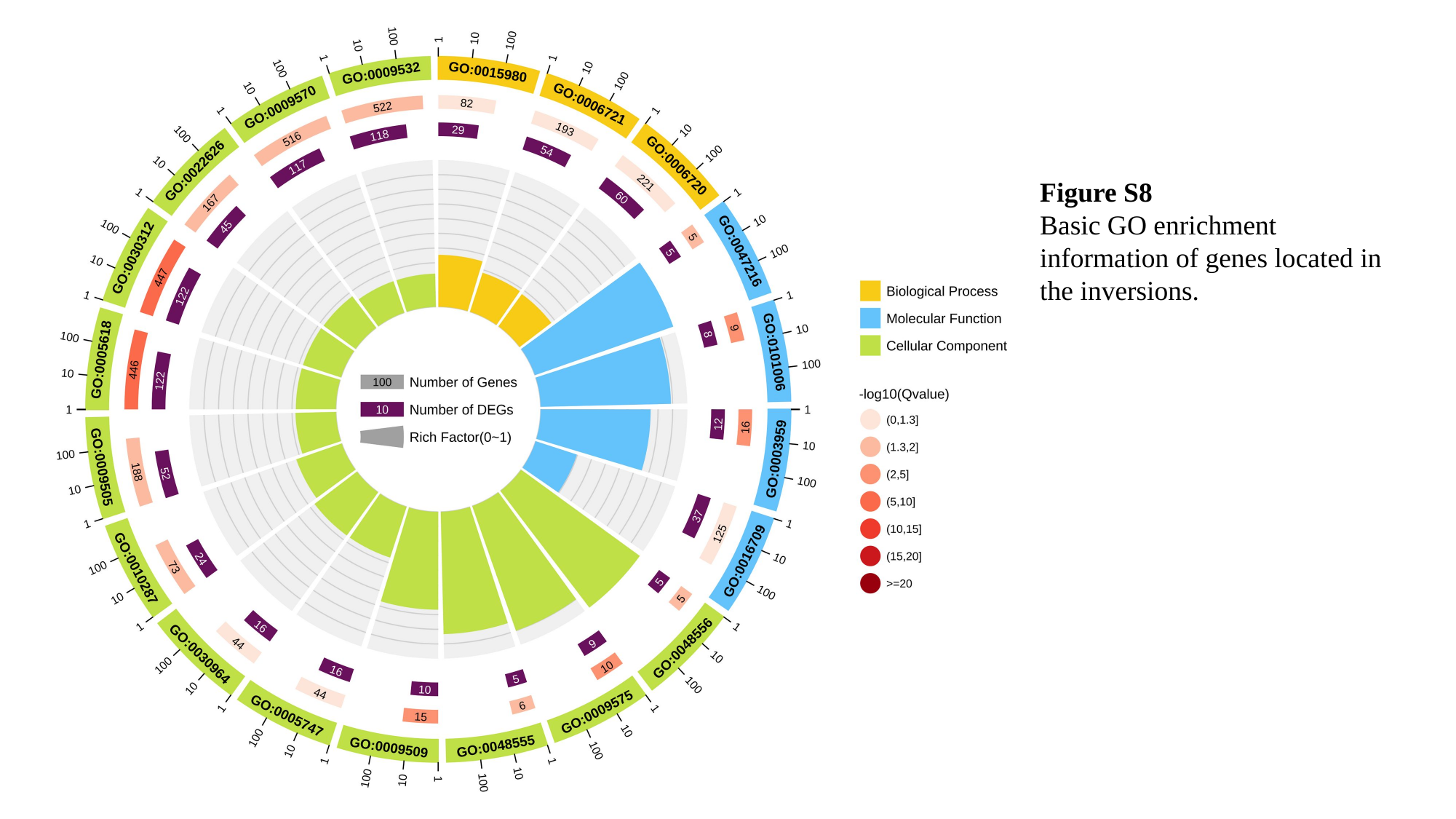

Figure S8
Basic GO enrichment information of genes located in the inversions.

## Slide 9
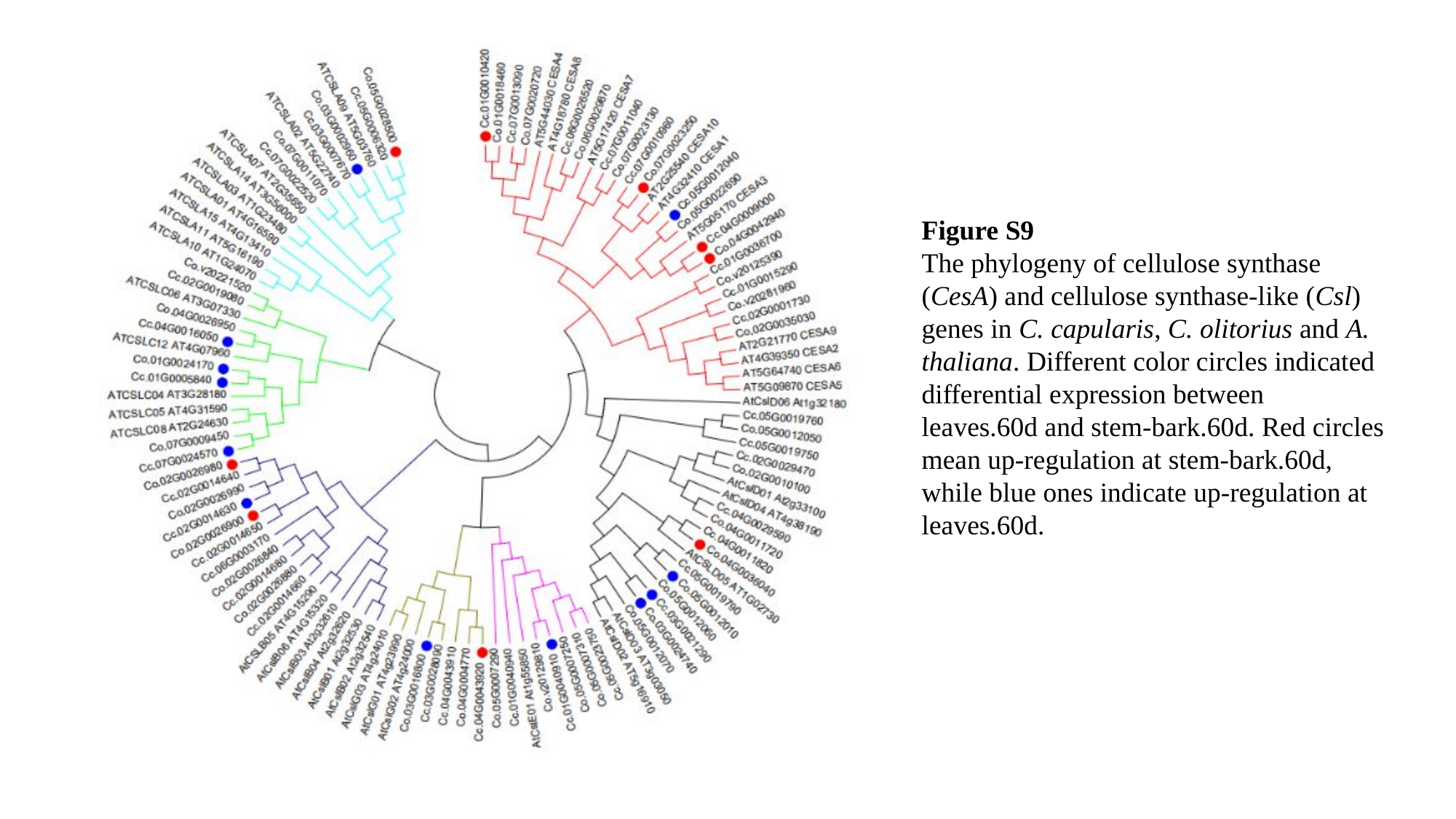

Figure S9
The phylogeny of cellulose synthase (CesA) and cellulose synthase-like (Csl) genes in C. capularis, C. olitorius and A. thaliana. Different color circles indicated differential expression between leaves.60d and stem-bark.60d. Red circles mean up-regulation at stem-bark.60d, while blue ones indicate up-regulation at leaves.60d.

## Slide 10
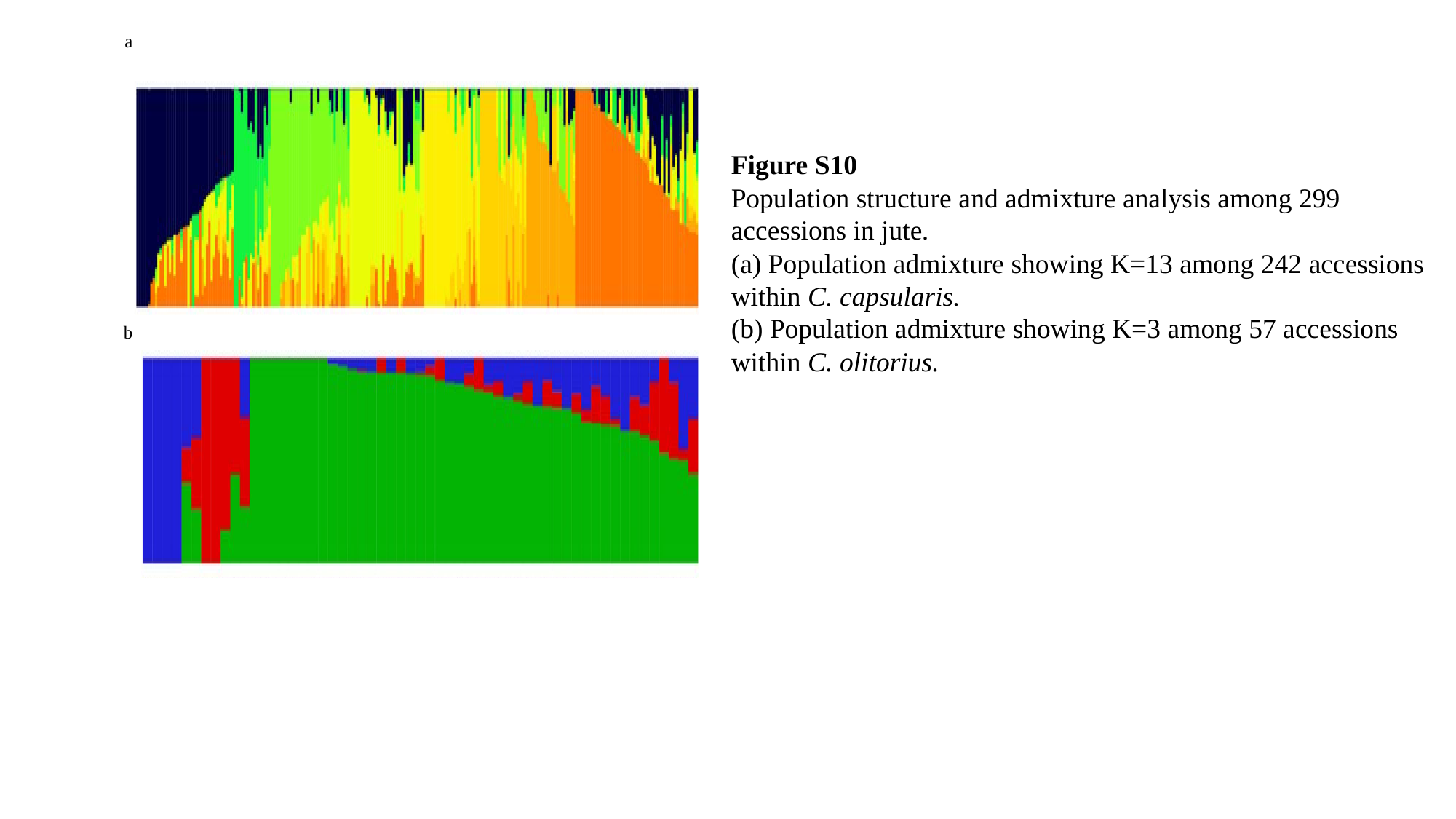

a
Figure S10
Population structure and admixture analysis among 299 accessions in jute.
(a) Population admixture showing K=13 among 242 accessions within C. capsularis.
(b) Population admixture showing K=3 among 57 accessions within C. olitorius.
b

## Slide 11
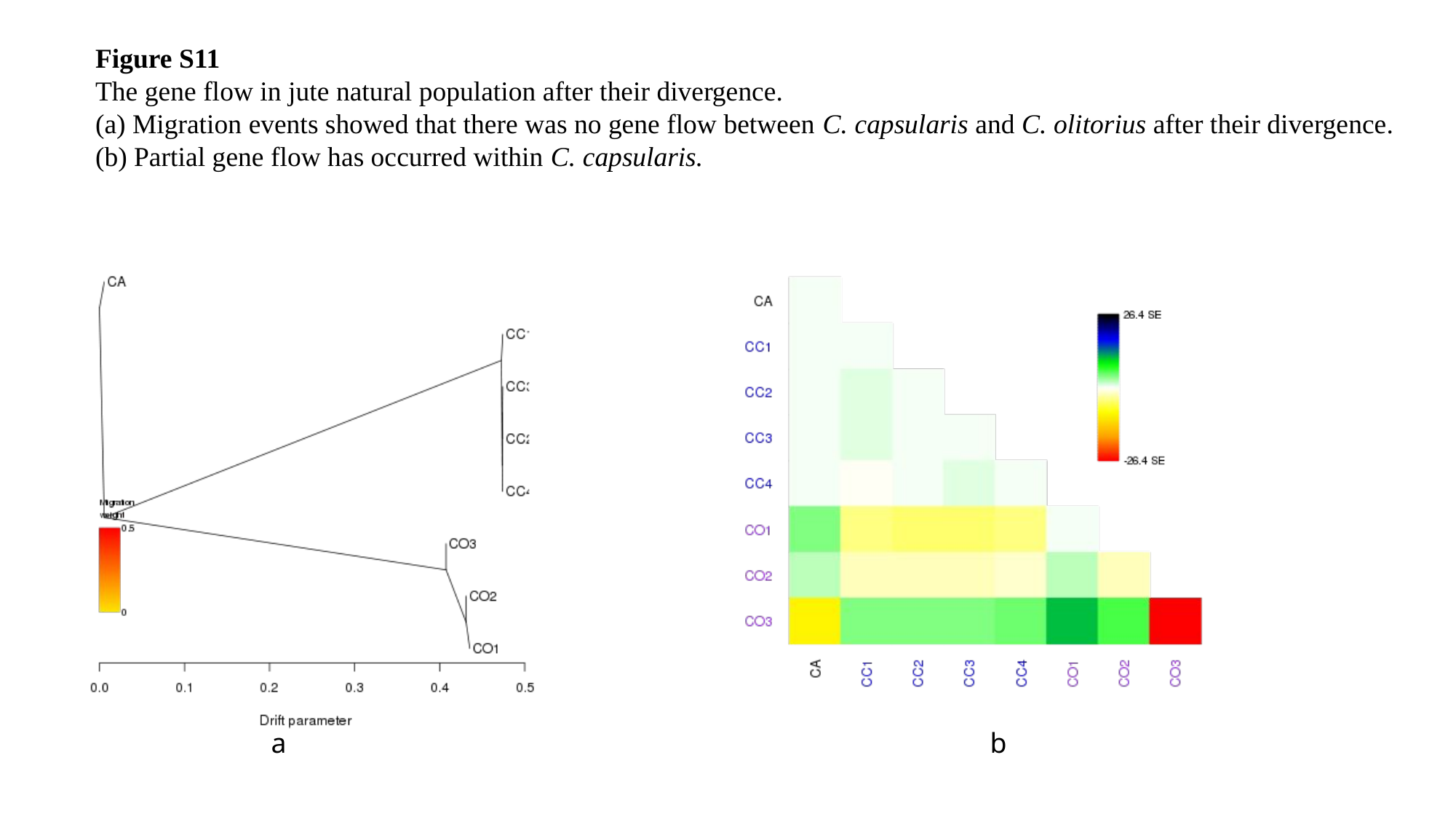

Figure S11
The gene flow in jute natural population after their divergence.
(a) Migration events showed that there was no gene flow between C. capsularis and C. olitorius after their divergence.
(b) Partial gene flow has occurred within C. capsularis.
a
b

## Slide 12
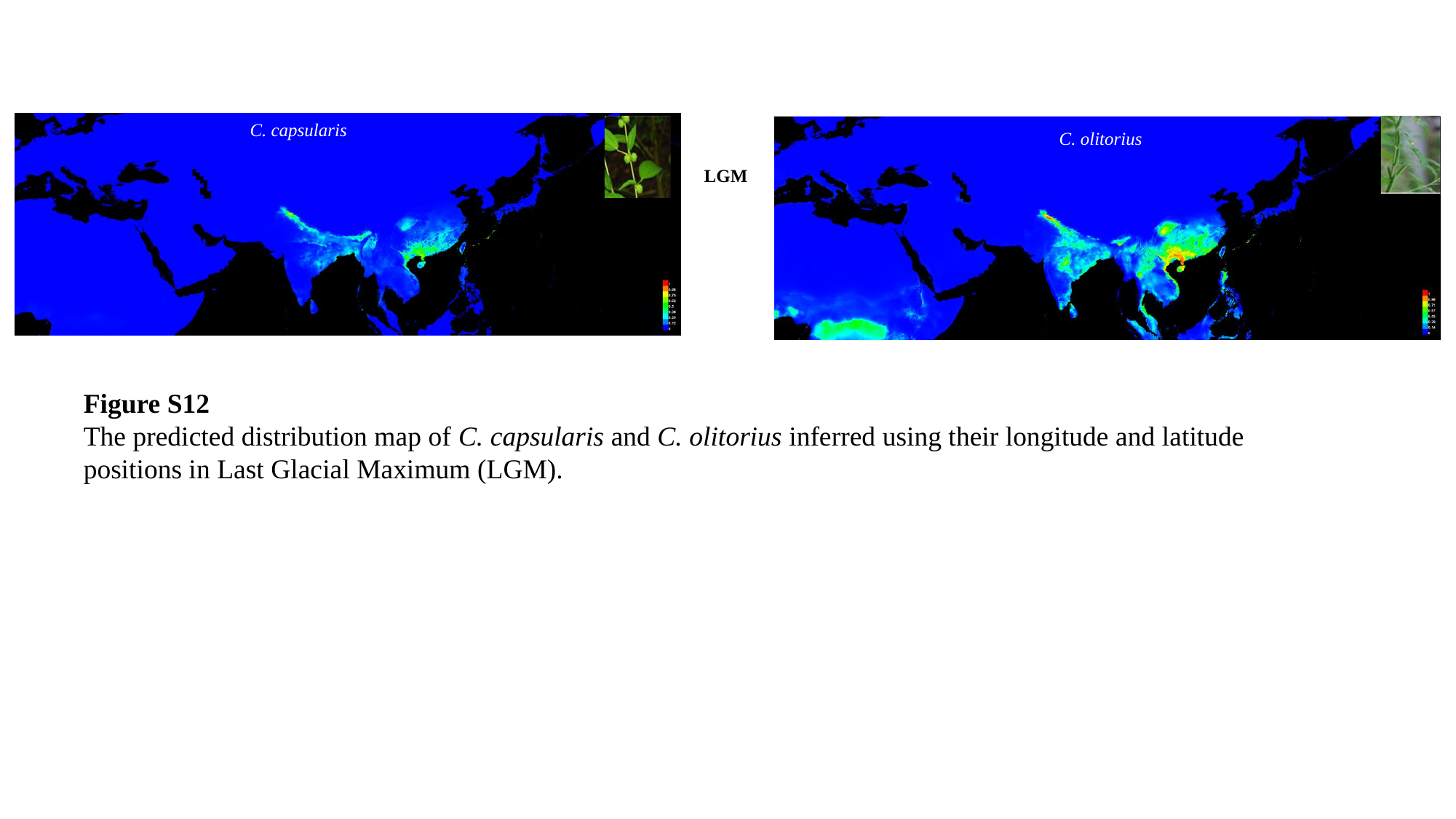

LGM
C. capsularis
C. olitorius
Figure S12
The predicted distribution map of C. capsularis and C. olitorius inferred using their longitude and latitude positions in Last Glacial Maximum (LGM).

## Slide 13
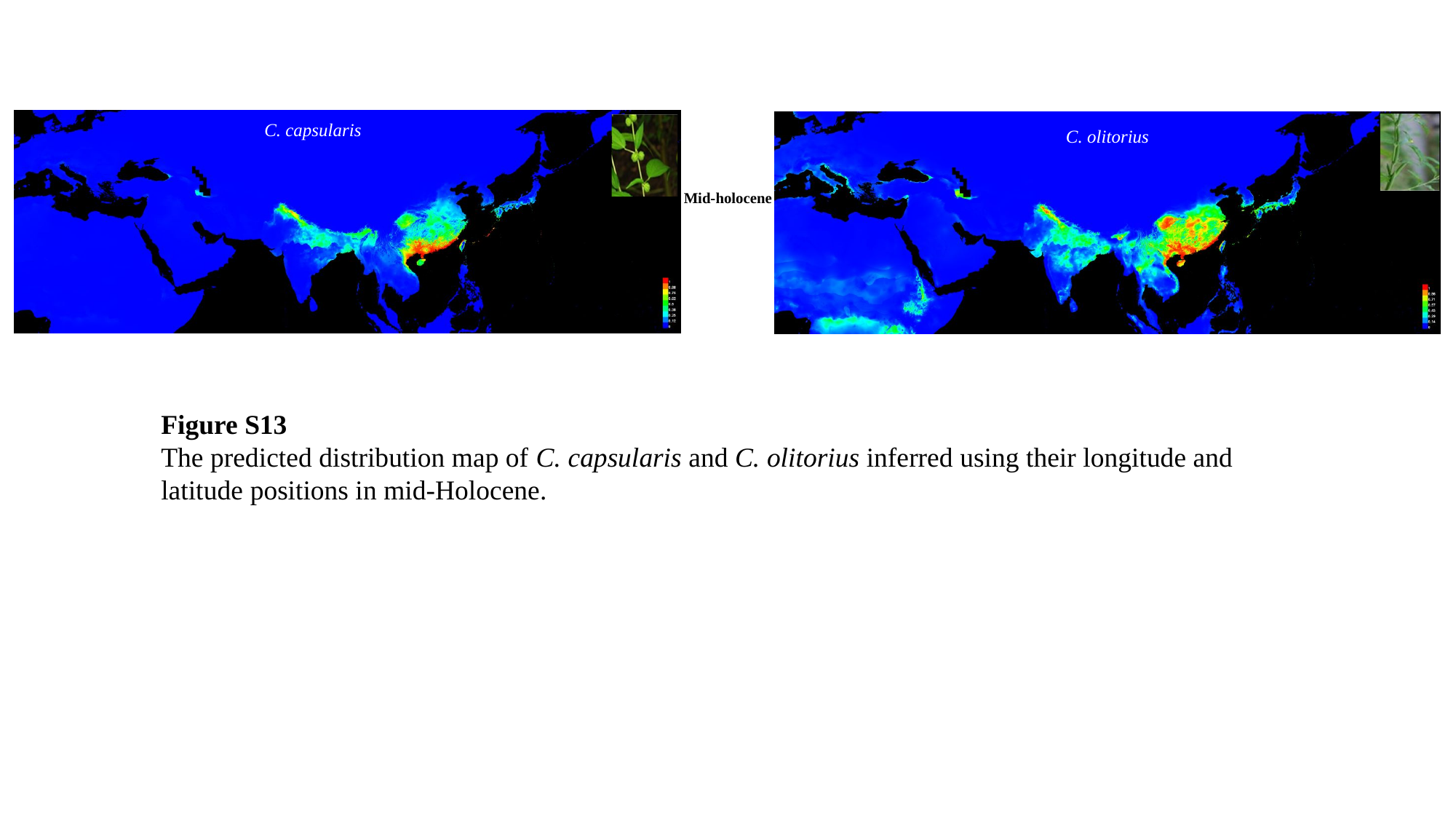

C. capsularis
C. olitorius
Mid-holocene
Figure S13
The predicted distribution map of C. capsularis and C. olitorius inferred using their longitude and latitude positions in mid-Holocene.

## Slide 14
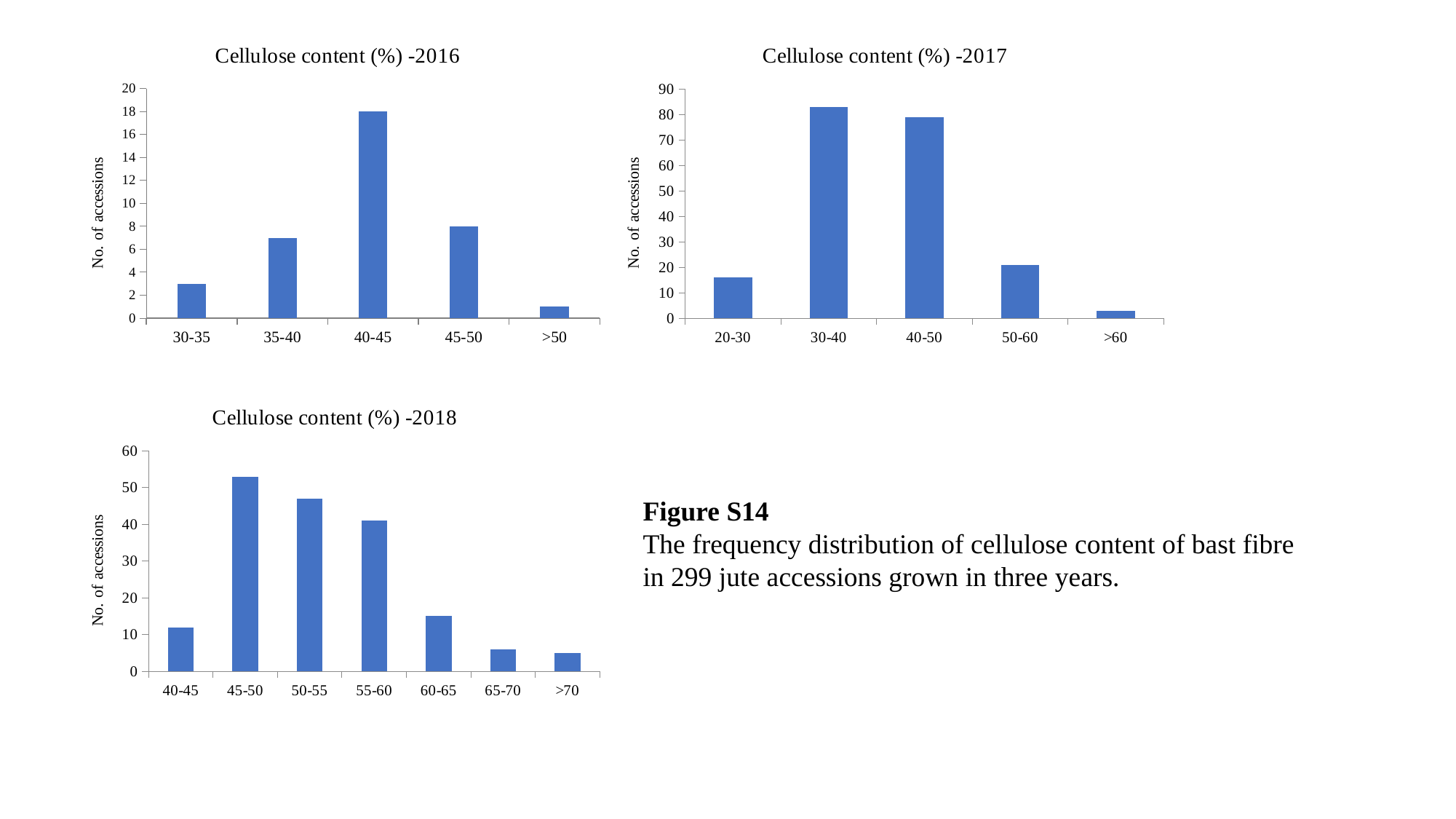

### Chart: Cellulose content (%) -2016
| Category | |
|---|---|
| 30-35 | 3.0 |
| 35-40 | 7.0 |
| 40-45 | 18.0 |
| 45-50 | 8.0 |
| >50 | 1.0 |
### Chart: Cellulose content (%) -2017
| Category | |
|---|---|
| 20-30 | 16.0 |
| 30-40 | 83.0 |
| 40-50 | 79.0 |
| 50-60 | 21.0 |
| >60 | 3.0 |
### Chart: Cellulose content (%) -2018
| Category | |
|---|---|
| 40-45 | 12.0 |
| 45-50 | 53.0 |
| 50-55 | 47.0 |
| 55-60 | 41.0 |
| 60-65 | 15.0 |
| 65-70 | 6.0 |
| >70 | 5.0 |Figure S14
The frequency distribution of cellulose content of bast fibre in 299 jute accessions grown in three years.

## Slide 15
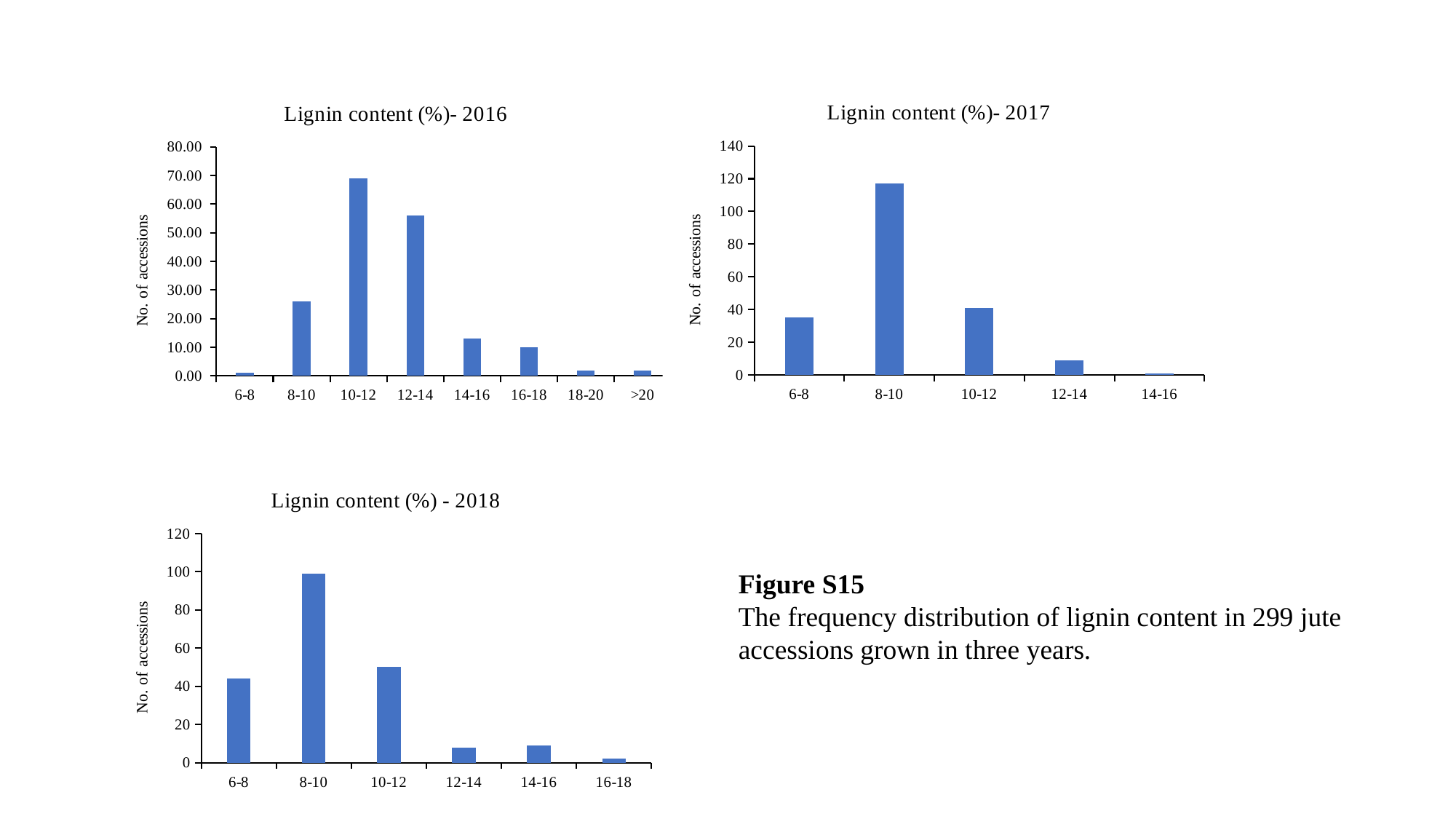

### Chart: Lignin content (%)- 2017
| Category | |
|---|---|
| 6-8 | 35.0 |
| 8-10 | 117.0 |
| 10-12 | 41.0 |
| 12-14 | 9.0 |
| 14-16 | 1.0 |
### Chart: Lignin content (%)- 2016
| Category | |
|---|---|
| 6-8 | 1.0 |
| 8-10 | 26.0 |
| 10-12 | 69.0 |
| 12-14 | 56.0 |
| 14-16 | 13.0 |
| 16-18 | 10.0 |
| 18-20 | 2.0 |
| >20 | 2.0 |
### Chart: Lignin content (%) - 2018
| Category | |
|---|---|
| 6-8 | 44.0 |
| 8-10 | 99.0 |
| 10-12 | 50.0 |
| 12-14 | 8.0 |
| 14-16 | 9.0 |
| 16-18 | 2.0 |Figure S15
The frequency distribution of lignin content in 299 jute accessions grown in three years.

## Slide 16
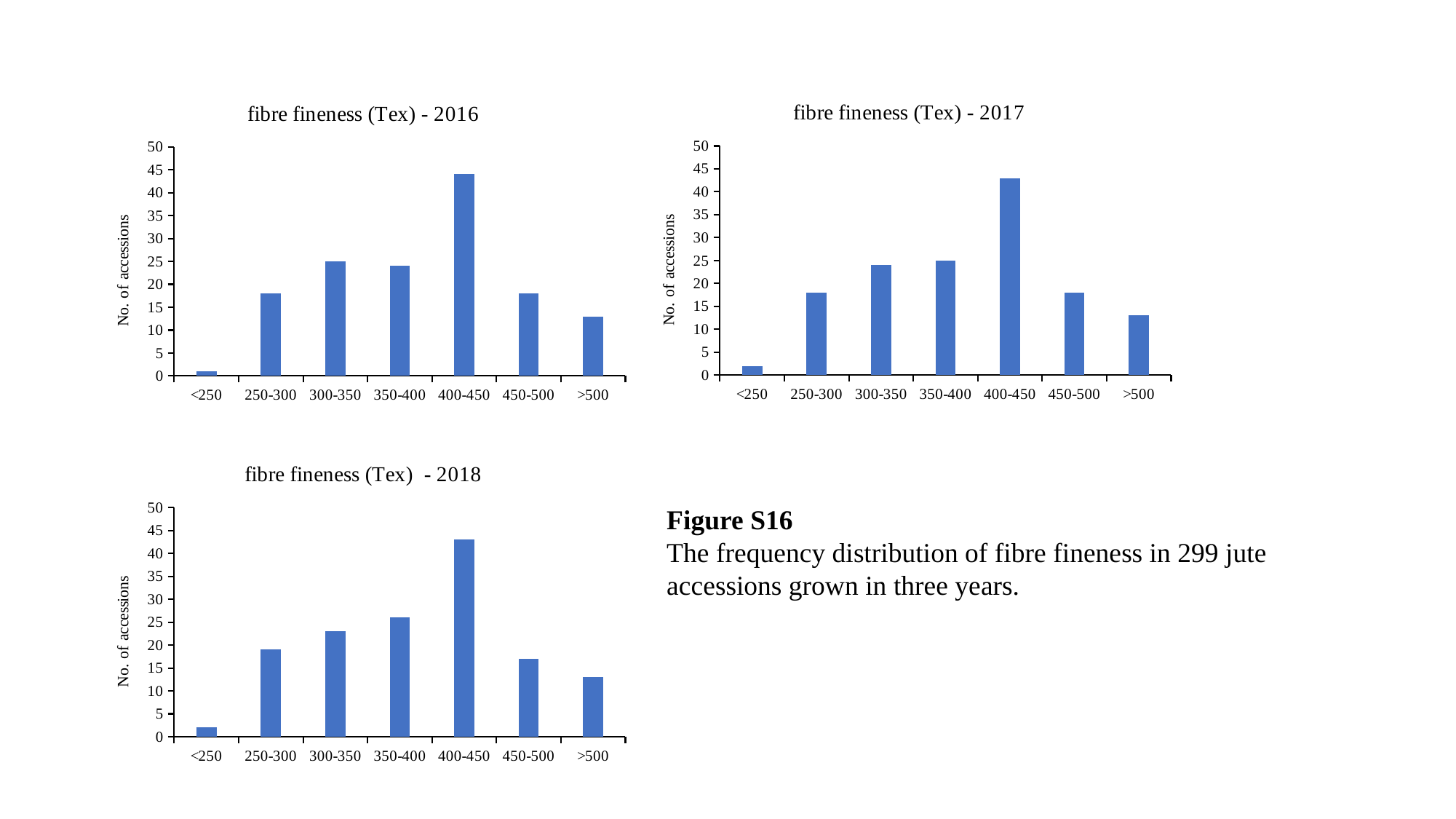

### Chart: fibre fineness (Tex) - 2017
| Category | |
|---|---|
| <250 | 2.0 |
| 250-300 | 18.0 |
| 300-350 | 24.0 |
| 350-400 | 25.0 |
| 400-450 | 43.0 |
| 450-500 | 18.0 |
| >500 | 13.0 |
### Chart: fibre fineness (Tex) - 2016
| Category | |
|---|---|
| <250 | 1.0 |
| 250-300 | 18.0 |
| 300-350 | 25.0 |
| 350-400 | 24.0 |
| 400-450 | 44.0 |
| 450-500 | 18.0 |
| >500 | 13.0 |
### Chart: fibre fineness (Tex) - 2018
| Category | |
|---|---|
| <250 | 2.0 |
| 250-300 | 19.0 |
| 300-350 | 23.0 |
| 350-400 | 26.0 |
| 400-450 | 43.0 |
| 450-500 | 17.0 |
| >500 | 13.0 |Figure S16
The frequency distribution of fibre fineness in 299 jute accessions grown in three years.

## Slide 17
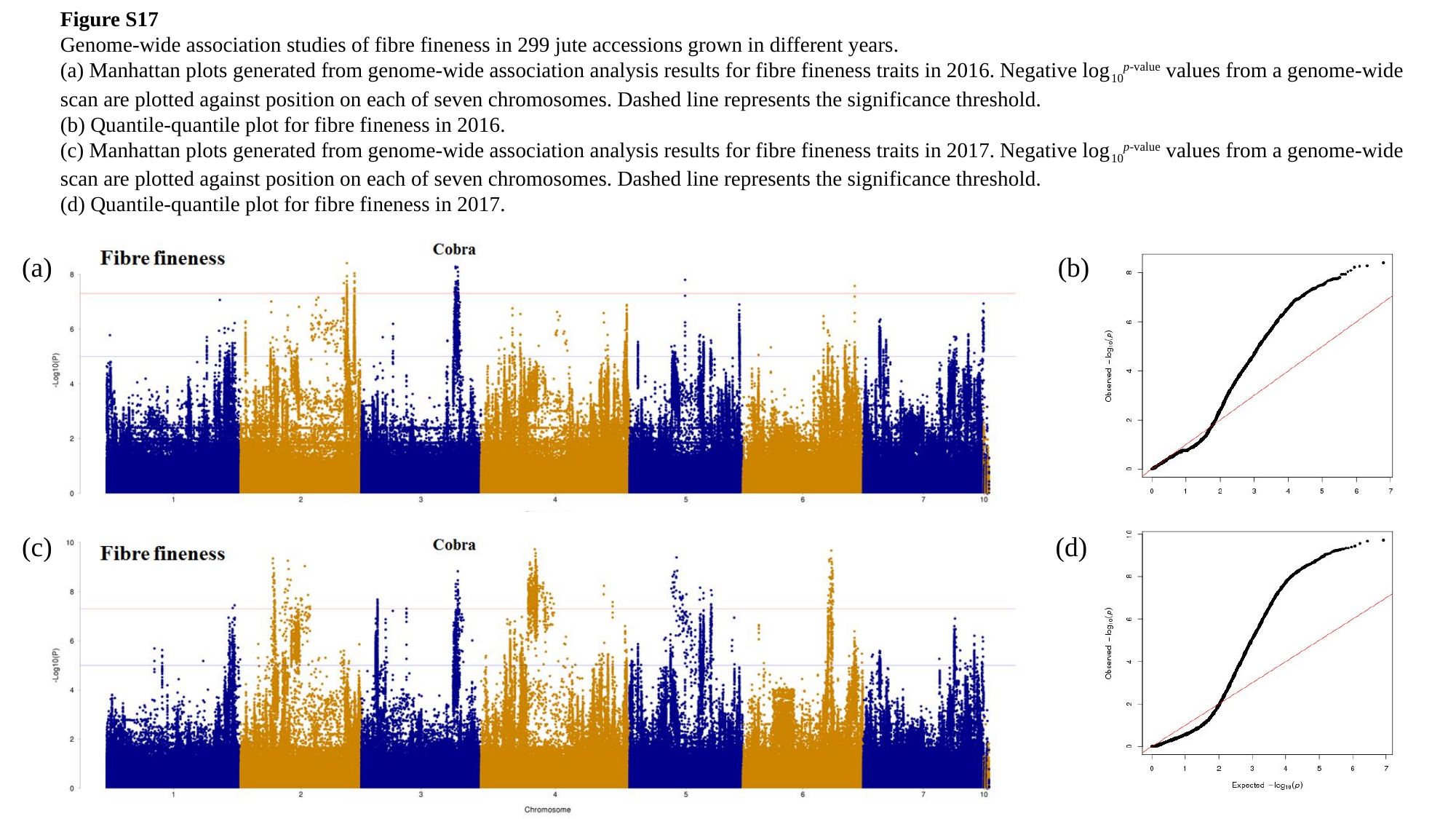

Figure S17
Genome-wide association studies of fibre fineness in 299 jute accessions grown in different years.
(a) Manhattan plots generated from genome-wide association analysis results for fibre fineness traits in 2016. Negative log10p-value values from a genome-wide scan are plotted against position on each of seven chromosomes. Dashed line represents the significance threshold.
(b) Quantile-quantile plot for fibre fineness in 2016.
(c) Manhattan plots generated from genome-wide association analysis results for fibre fineness traits in 2017. Negative log10p-value values from a genome-wide scan are plotted against position on each of seven chromosomes. Dashed line represents the significance threshold.
(d) Quantile-quantile plot for fibre fineness in 2017.
(a)
(b)
(c)
(d)

## Slide 18
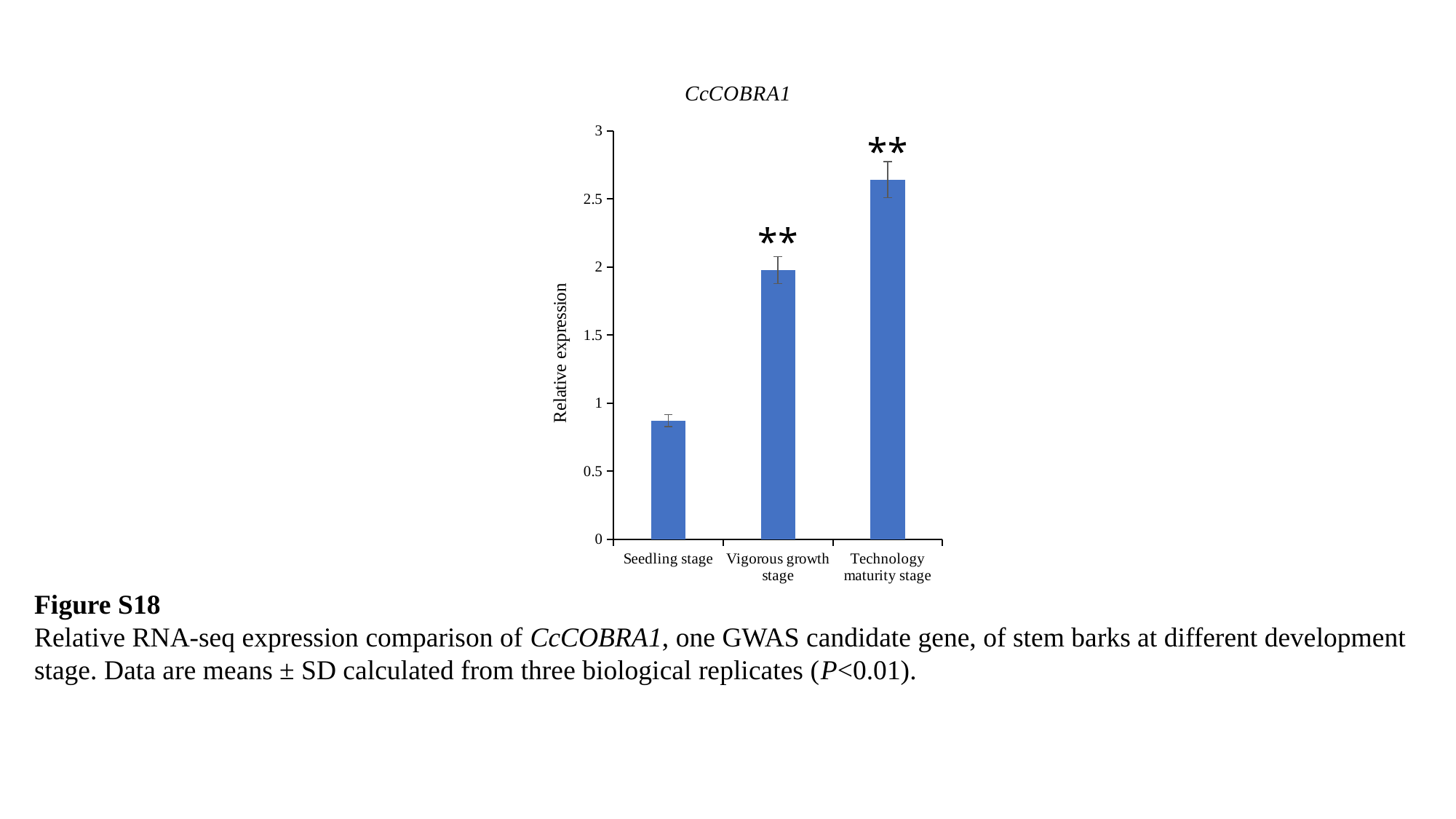

### Chart: CcCOBRA1
| Category | CcCOBRA |
|---|---|
| Seedling stage | 0.8718436485093177 |
| Vigorous growth stage | 1.9781956296816519 |
| Technology maturity stage | 2.64154602908752 |Figure S18
Relative RNA-seq expression comparison of CcCOBRA1, one GWAS candidate gene, of stem barks at different development stage. Data are means ± SD calculated from three biological replicates (P<0.01).

## Slide 19
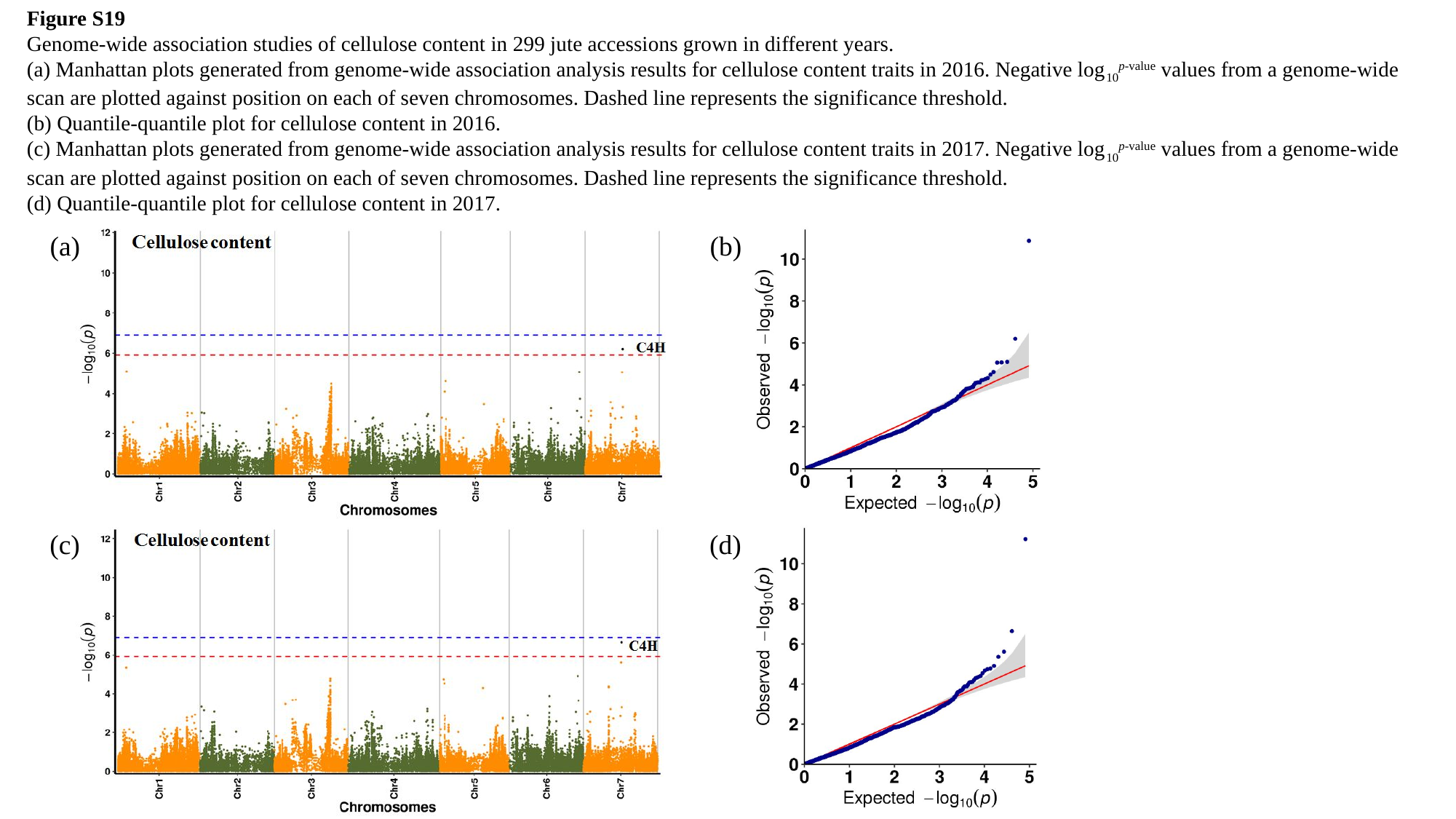

Figure S19
Genome-wide association studies of cellulose content in 299 jute accessions grown in different years.
(a) Manhattan plots generated from genome-wide association analysis results for cellulose content traits in 2016. Negative log10p-value values from a genome-wide scan are plotted against position on each of seven chromosomes. Dashed line represents the significance threshold.
(b) Quantile-quantile plot for cellulose content in 2016.
(c) Manhattan plots generated from genome-wide association analysis results for cellulose content traits in 2017. Negative log10p-value values from a genome-wide scan are plotted against position on each of seven chromosomes. Dashed line represents the significance threshold.
(d) Quantile-quantile plot for cellulose content in 2017.
(a)
(b)
(c)
(d)

## Slide 20
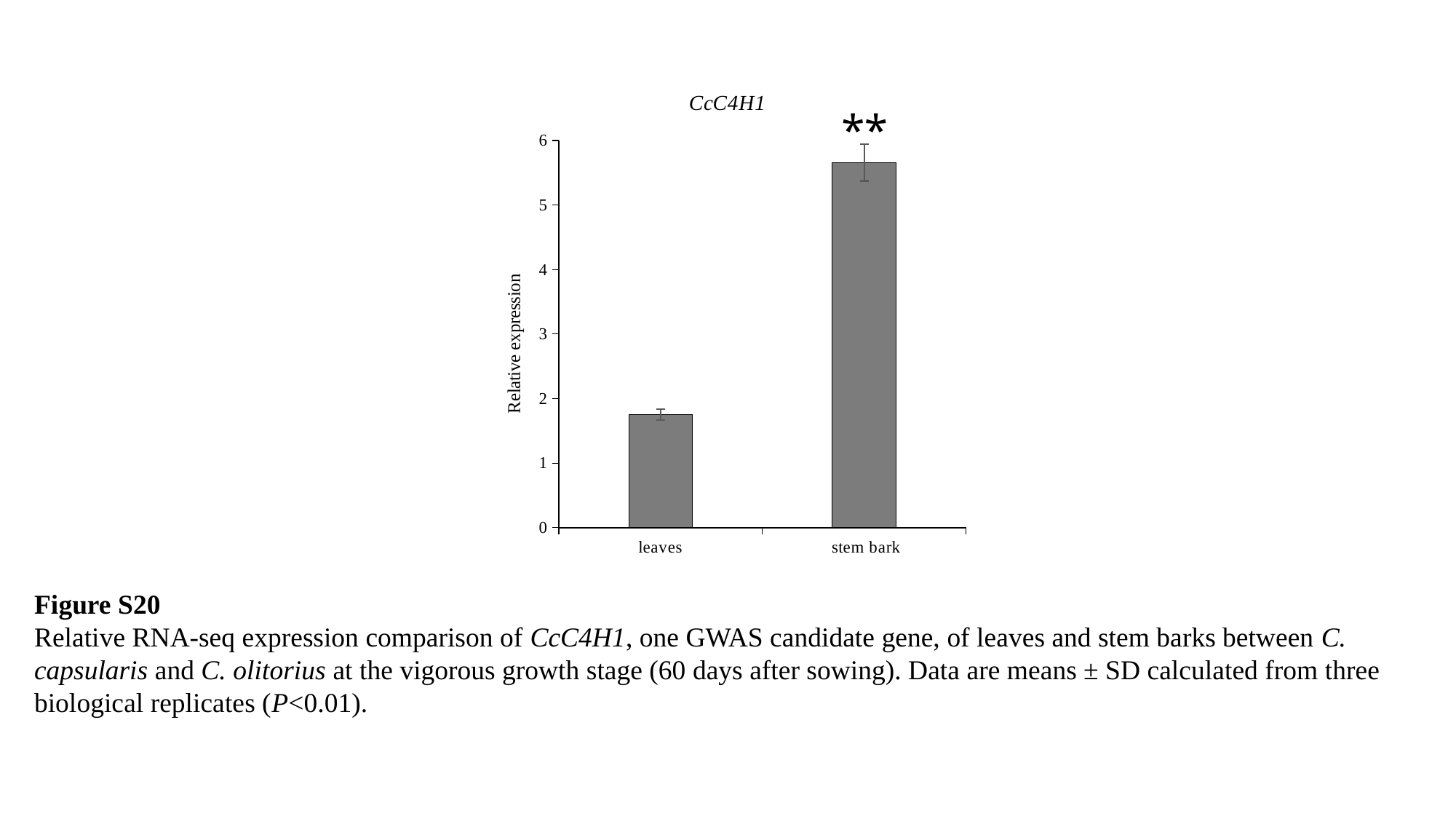

### Chart:
| Category | CcC4H1 |
|---|---|
| leaves | 1.752748591407134 |
| stem bark | 5.656210320182599 |Figure S20
Relative RNA-seq expression comparison of CcC4H1, one GWAS candidate gene, of leaves and stem barks between C. capsularis and C. olitorius at the vigorous growth stage (60 days after sowing). Data are means ± SD calculated from three biological replicates (P<0.01).

## Slide 21
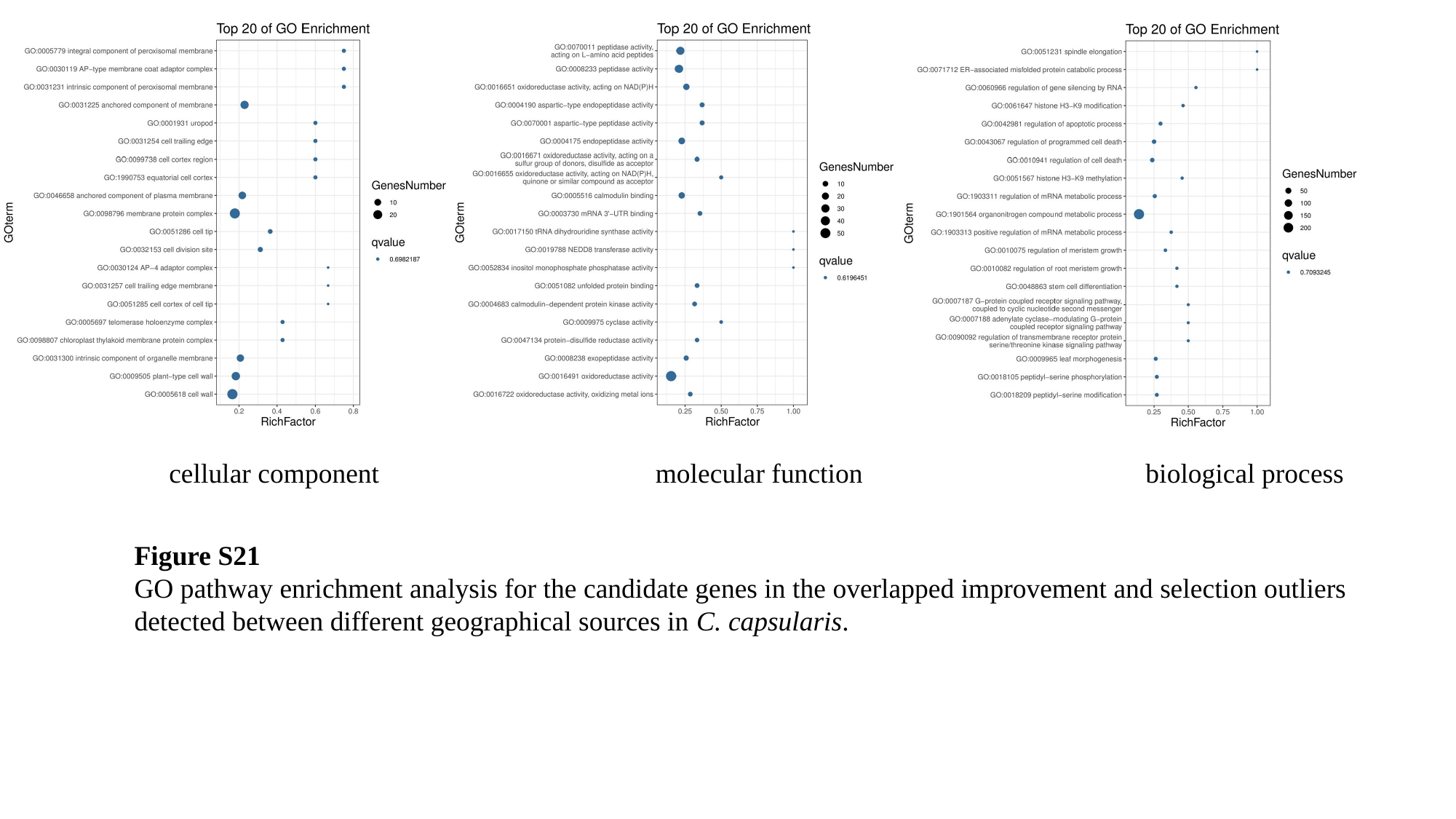

cellular component molecular function biological process
Figure S21
GO pathway enrichment analysis for the candidate genes in the overlapped improvement and selection outliers detected between different geographical sources in C. capsularis.

## Slide 22
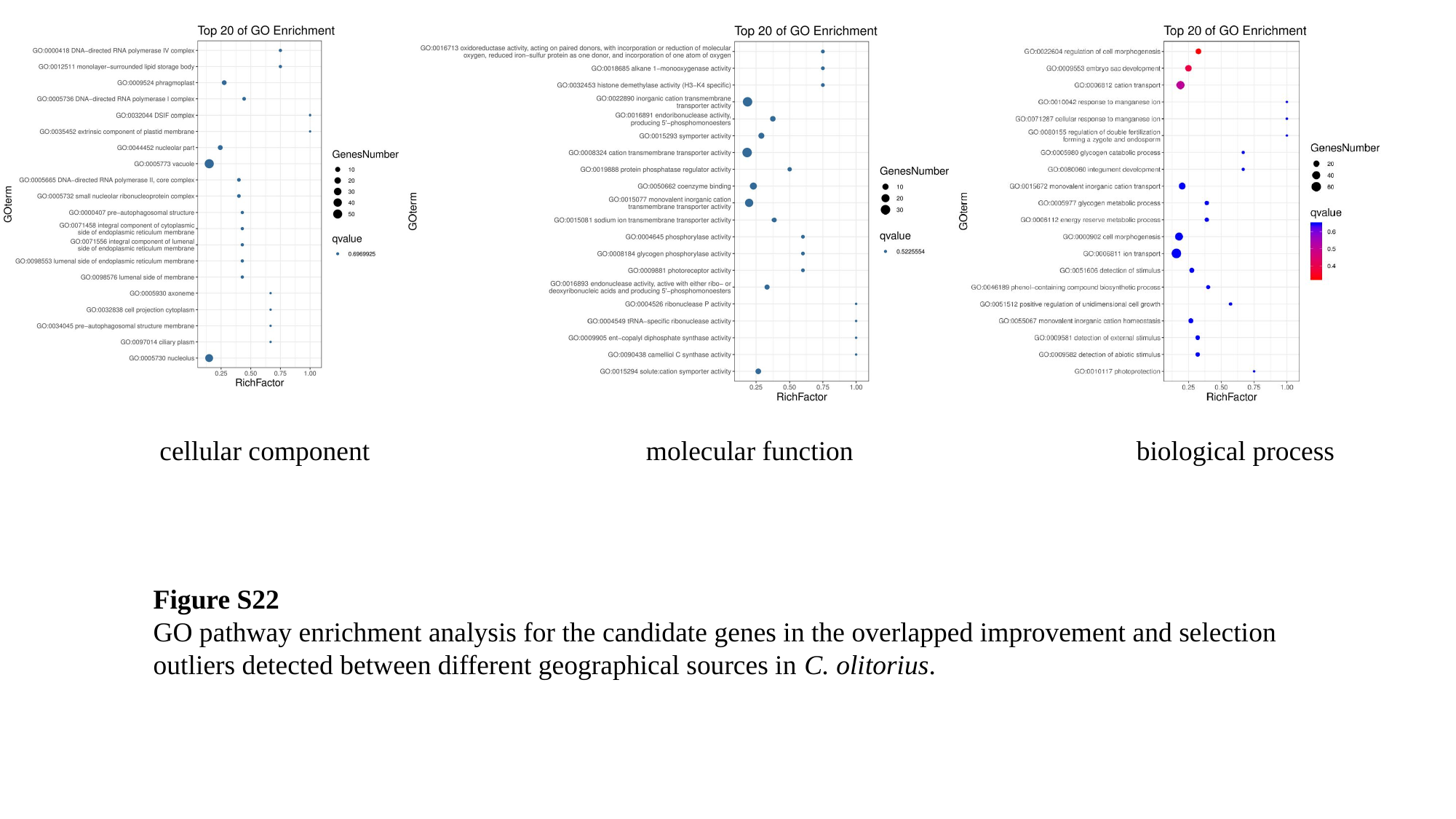

cellular component molecular function biological process
Figure S22
GO pathway enrichment analysis for the candidate genes in the overlapped improvement and selection outliers detected between different geographical sources in C. olitorius.
