# Supplementary material for: Reference genomes of the two cultivated jute species
Source: Plant Biotechnol J. 2021 Jul 8;19(11):2235–48. doi: 10.1111/pbi.13652 (PMC8541789; doi:10.1111/pbi.13652)
Supplement: Supplementary file 3 — Appendix S1 Materials and Methods. [file PBI-19-2235-s003.docx]

**Experimental procedures**

**1** **Genome sequencing and assembly**

**1.1 Sample collection**

*C. capsularis* var. Huangma 179 and *C. olitorius* var. Kuanyechangguo, which are the control cultivars in the official variety registry in China, are chosen for reference genome sequencing and assembly. The plants were grown at the farm of Fujian Agriculture and Forestry University, Fuzhou, China. And leaves were collected for genome sequencing from a single plant. Samples from different stages and tissues were collected for RNA sequencing, including hypocotyls, roots, stem barks, stem sticks, leaves, and buds; fiber quality investigations. Three samples from each tissue were immediately frozen in liquid nitrogen and stored at -80°C separately as three biological replicates for the following RNA isolation.

**1.2 DNA extraction and library preparation**

Genomics DNA from the two species of jute was extracted from leaf tissue of individuals, and were subjected to 500-bp paired-end (PE) libraries construction using the NEBNext Ultra DNA Library Prep Kit for Illumina sequencing. Sequencing was performed using the Illumina HiSeq X10 platform (Illumina, San Diego, CA, USA), which were conducted at the Center for Genomics and Biotechnology (Fuzhou, China) and Beijing Biomarker Technology Co., Ltd (Beijing, China). Trimmomatic v0.36(Bolger et al., 2014) software was used to trimmed 150 bp PE reads with default parameters. For the Pacbio sequencing, DNA was extracted from leaf tissue of same single plant at Genomics and Biotechnology (Fuzhou, China) and the Next genomics Technology Co., Ltd (Wuhan, China). More than 50 µg of sheared and concentrated DNA was applied to size-selection by the BluePippin system. A total of 41 Gb each of sequence data was generated for both the *C. capsularis* and *C. olitorius* genome.

**1.3 RNA extraction and library preparation**

RNA was extracted using the Illumina TruSeq™ RNA Sample Preparation Kit (Illumina, San Diego, CA, USA) following the manufacturer’s recommendations (Kircher et al., 2009). 2µg of total RNA was processed using the TruSeq RNA Sample Preparation kit (Illumina) for RNA-seq libraries construction. It was followed by sequencing on the Illumina HiSeq ×10 platform at Center for Genomics and Biotechnology (Fuzhou, China) and Beijing Novogene Biological Information Technology Co., Ltd. (Beijing, China).

**1.4 Genome assembly**

CANU package (Koren et al., 2017) version 1.5 was used for contig assembly and self-correction, and where assembled fallowing these parameters; corOutCoverage=100, ovbMemory=8g, maxMemory=500g, maxThreads=48, ovsMemory=8-500g, ovsThreads=4, oveMemory=32g on a SGE grid. Subsequently, the draft assembly was polished using Quiver (https://github.com/PacificBiosciences/GenomicConsensus). Illumina short reads were recruited to increases the accuracy of assembly, and for further polishing using the Pilon program, we generated 340 Mb and 394 Mb genome assembly, respectively. We also assessed the completeness of the assemblies using the CEGMA v2.5 and BUSCO version 3(Simao et al., 2015) pipelines.

**1.5 HiC sequencing and scaffolding**

Hi-C libraries were created from tender leaves of the plants in BioMarker Technologies Company as described (Xie et al., 2015). Briefly, the leaves were fixed with formaldehyde, lysed, and then the cross-linked DNA digested with *HindIII* overnight. Sticky ends were biotinylated and proximity-ligated to form chimeric junctions, that were enriched for and then physically sheared to a size of 500-700 bp. Chimeric fragments representing the original cross-linked long-distance physical interactions were then processed into paired-end sequencing libraries, 148 and 239 million of 150 bp paired-end Illumina reads were produced, respectively. The paired-end reads were uniquely mapped onto the draft assembly contigs, and we constructed 7 chromosome clusters using the HiC data in *Cc* and *Co* draft genome, scaffolding performed using LACHESIS program (Paritosh et al., 2020).

**2** **Transcriptome sequencing and gene annotation**

**2.1 Assembly of RNA-seq data**

Selected RNA-seq samples were imported into Trinity de novo assembly and genome-guided assembly pipelines with default parameters (Haas et al., 2013). RSEM was used to calculate transcript abundance (Li and Dewey, 2011). Transcripts with FPKM < 1 and iso-percentage < 3 % were removed from further analysis. The filtered transcripts were subject to the PASA program for the construction of the complete transcripts. PASA (Brian J. Haas et al., 2003) can take advantage of the high sensitivity of reference-based assembly while leveraging the ability of *de novo* assembly to detect novel transcripts.

**2.2 Annotation of protein-coding genes**

The PASA-assembled transcripts described above were used for training. The nearly “full-length” transcripts were evaluated by comparing with the UniProt plant protein database and proteins that were covered at least 95 % were retained as candidates. Then *ab initio* gene predictors, including SNAP (Korf, 2004), GENEMARK (Lomsadze et al., 2005), and AUGUSTUS (Stanke et al., 2006), were each trained with those selected proteins.

After that, the MAKER pipeline was used to integrate multiple tiers of coding evidence, including *ab initio* gene prediction, transcript evidence, and protein evidence, and generate a comprehensive set of protein-coding genes. Finally, a total of 25874 gene models were predicted for *Cc* genome and 28479 gene models for the *Co* genome. The assessment software BUSCO detected 86.1% and 90.8% of complete gene models in two species.

**2.3 Identification of the repetitive elements**

We first customized a *de novo* repeat library of the genome using RepeatModeler (http://www.repeatmasker.org/RepeatModeler/), which can automatically execute two *de novo* repeat finding programs, including RECON (version 1.08) (Bao and Eddy, 2002) and RepeatScout (version 1.0.5) (Price et al., 2005). The consensus TE sequences generated above were subject to RepeatMasker (version 4.05) to identify and cluster repetitive elements. Unknown TEs were further classified using TEclass (version 2.1.3) (Abrusan et al., 2009).

To identify tandem repeats within the genome, the Tandem Repeat Finder (TRF) package (version 4.07) was used with the modified parameters of “1 1 2 80 5 200 2000 –d -h” to find high order repeats.

**2.4 Gene expression analysis**

The heatmaps were created by R language based on the transformed data of log_2_ ^(FPKM+1)^ values. The FPKM (reads per kb per million reads) value was calculated for each gene. Differential gene expression analysis using edgeR. FDR (False Discovery Rate) <0.05 and log2(fold change)>1 were identified as significantly up-regulated gene. FDR <0.05 and log2(fold change) <1 were set as the threshold for the significant down-regulated gene. Three independent reactions were performed for putative genes using the Fast Start Universal SYBR^®^ Green Master (ROX) with a 7500 real-time PCR machine (ABI) referring to manufacturer’s directions. Each reaction was carried out in three biological replicates and the relative gene expression was determined with 2^–△△CT^ method. Three biological repeats with two technical replicates of each sample were performed to acquire reliable results. Data are the mean ± standard deviation of three independent experiments. Error bars indicate standard deviation of the means. All data analyses were conducted using SPSS Statistics 20.

**3 Differentially expressed genes (DEGs) analysis**

The FPKM (reads per kb per million reads) value was calculated for each gene. Differential gene expression analysis using edgeR. FDR (False Discover Rate) <0.05 and log_2_(fold change)>0 were identified significantly up-regulated gene. FDR <0.05 and log_2_(fold change) <0 were set as the threshold for significant down-regulated gene.

**4 Genome comparison and evolution**

**4.1 Phylogenetic analysis**

The single-copy genes of each species were identified using the PYTHON script base on the OrthoMCL clusters and performed multiple alignments of protein sequences with MUSCLE (Edgar, 2004). Divergence times between *C. capsularis*, *C. olitorius* and other species were estimated using MEGA (Tamura et al., 2011).

**4.2 Genome speciation event deduced from orthologous pairs**

The orthologous pairs were selected base on the OrthoMCL clusters, which were used to calculate the Ks (the number of nonsynonymous substitutions per nonsynonymous site) by YN00 in the PAML package.

**5 Jute population genomics**

**5.1 Plant materials and phenotyping**

The association population used in this study consisted of 300 diverse jute accessions, of which 242 are *C. capsularis*, and 57 are *C. olitorius*. These accessions were originated from different countries all around the world and maintained in the Key Laboratory for Genetics, Breeding and Multiple Utilization of Crops, Ministry of Education, Fujian Agricultural and Forestry University. They broadly represent the genetic diversity of the jute gene pool. All accessions evaluated in this study were planted in a randomized complete block design with three replications in field trials in Fuzhou, Fujian, China (26°08′ N, 119°30′ E) over three years (2016, 2017 and 2018). And the essential agronomic traits were evaluated.

**5.2 Variants calling and annotation**

In total, 300 jute accessions were resequenced for population genomics analyses. The raw pair-end reads were trimmed to remove the adaptors and low-quality bases using Trimmomatic (Bolger et al., 2014) after quality control by FastQC. The reads were filtered with a sliding window of size 7, with an average Phred score scale = 20 within the window. The trimmed reads were mapped to the reference genome using Bowtie2(Langmead and Salzberg, 2012) with default parameters. The mapped reads were sorted, and duplicated reads were removed using SAMtools (Li et al., 2009). The Realigner Target Creator and Indel Realigner programs from the Genome Analysis Toolkit (GATK) package (McKenna et al., 2010) were used for global realignment of reads around indels from the sorted BAM files. The Haplotype Caller of the GATK was used to estimate the SNPs for putative diploids using the default parameters. The distribution of calling depths (DP) of each raw variant was calculated as a criterion for variant filtering. Low depths and repetitive variants were removed from the raw VCF file if they had DP < 2 or DP > 40, minQ < 20. We allowed the variant sites with the maximum-missing rate as 20%. These filtering strategies reduced the raw, unfiltered variants to the working set of 3,415,772 variants (SNPs). SnpEff v3.6c was used to assign variant effects based on gene models from reference genome annotation. A total of 3,415,772 SNP markers were used for the subsequent analysis.

**5.3 Genome-wide genetic diversity and LD decay estimation**

The high confidence filtered 3,415,772 variant set was used for population genomics statistics estimations. Population statistics of SNP density, π, and Tajima’s D were calculated from the filtered VCF file in a 200-kb sliding window for π, SNPs density, Tajima’s D and *F*-statistics (Weir & Cockerham *Fst*) in VCFtools (Danecek et al., 2011). A One-sample t-test was used to evaluate the statistically significant if Tajima’s D is deviating from zero. Genome-wide Linkage disequilibrium (LD) was calculated, and the LD decay curve was fitted in PopLDdecay (https://github.com/BGI-shenzhen/PopLDdecay) with default parameters.

**5.4 PCA, phylogeny and population structure**

A map of the geographic positions of 300 accessions gathered from all around the world was generated using the R software package ‘map tools’ (http://r-forge.r-project.org/projects/maptools/). Principal component analysis (PCA) was performed using the GCTA software on the filtered 3,415,772 variants. The input Plink binary files are transformed from the filtered VCFs file using VCFtools (Danecek et al., 2011) and PLINK (Purcell et al., 2007). The top two principal components were used for assigning the 300 accessions. Bi-allelic and polymorphic 3,415,772 SNPs were used for reconstructing the phylogenetic relationships among 300 accessions using SNPhylo (Lee et al., 2014) software. Before tree construction, we filtered and pruned the SNPs (with MAF< 0.05, and missing rate > 0.3, LD threshold = 0.1). The multiple consensus sequences were aligned using MUSCLE (Edgar, 2004). ML trees were constructed using the maximum likelihood method by running DNAML programs in the PHYLIP package. Besides, the BS tree was constructed by bootstrapping (bootstrap =10000) analysis using PHANGORN package (Schliep, 2011). Figtree v.1.4 (http://tree.bio.ed.ac.uk/software/figtree/) was used to visualize the trees. The optimal ancestral population stratification was estimated from the same variants set with ADMIXTURE (Alexander et al., 2009) using ancestral population clusters K = 1~20. The smallest population was selected for cross-validation error. DISTRUCT (Rosenberg, 2004) was used to plot the population stratification results for K = 1 through K = 20.

**5.5 Demography history of effective population size (*Ne*)**

Site frequency spectrum (SFS) of 300 cultivated accessions were estimated using ANGSD (Korneliussen et al., 2014). We calculated the site allele frequency likelihood based on the SAMTools (Li et al., 2009) genotype likelihood model at all sites. We then obtained a maximum likelihood estimate of the SFS using the Expectation-Maximization (EM) algorithm. The SFS was then used for estimating the population demography history using software Stairway plots (Liu and Fu, 2015) with 200 bootstrap iterations. Because of the variation of molecular substitution rate for wild jute and relative’s populations, we used a range of 1.5e-9, 6.5e-9, and 10e-9 as the mutation rate parameters for analysis. As the generation time of wild jute is one year, we carried out the estimation using one-year generation time when doing Stairway plots.

**5.6 Genome-wide association study (GWAS)**

The best linear unbiased prediction (BLUP) for each investigated trait of each accession was obtained using an R script, based on a linear model and corresponding characteristics with three replicates over three years. The resulting values were used as phenotypes for the association analysis. The multi-locus random-SNP-effect mixed linear model was used to test trait-SNP associations in mrMLM; PCA and K were controlled as fixed and random effects, respectively. The valid number of independent SNPs was calculated using GEC software (Browning and Browning, 2016). Only LD blocks containing at least one significant and one suggestive SNPs will be regarded as substantial loci for a robust considerable association signal in the GWAS.

**5.7 Genomic-wide selective sweeps scanning**

In terms of genomic-wide selection, we detected the absolute selective sweeps in the jute population with a grid size of 20 k using the SweeD (Pavlidis et al., 2013) software. The CLR (composite likelihood ratio) statistic was used as the criteria of recent selective sweeps by significant deviating from the neutral site frequency spectrum (SFS). The SFSs were calculated using ANGSD (Korneliussen et al., 2014). The identification of candidate sweeps regions by the CLR scores is scanned across the jute reference genome with each chromosome dividing into 2,000 windows in SweeD. The candidate sweep regions were identified as those exceeding the top 1% of CLR scores that significant deviations from neutrality. Genes overlapping sweep regions (including ±2kb flanking regions of each boundary) were treated as genes putatively under selection. The swept genes were then performed the functional GO annotation and enrichment analysis in Blast2Go v4.1(Conesa et al., 2005). We used swept genes as tested gene sets, the whole gene models of jute as references. The significance of enrichments was valued using the Fisher’s exact test.

**6 Overexpression of *CcCOBRA1***

*CcCOBRA1* was introduced into *Arabidopsis thaliana* and *CcCOBRA1*-overexpressing plants and wild type (WT) plants were obtained. Transgenic and WT plants grown in a transplanting box were grown under normal conditions. The expression of *CcCOBRA1* in vitro-grown transgenic and WT plants under normal conditions was measured by qRT-PCR. The cellulose content in leaves was measured by ultraviolet visible spectrophotometer.

**7 Development of Kompetitive Allele Specific PCR (KASP) marker**

Genotyping reaction reagents were purchased from the LGC, Biosearch Technologies (Beverly, MA, USA). A total of one KASP assay was developed and validated on the DNA of 299 individuals and two non-template controls (NTC). Assays were tested in 96-well formats with reaction set up of 10 µL [2 µL wet DNA (50 ng/µL final concentration of DNA)], 5 µL of KASP master mix, 0.5 µL of assay mix, and 2.5 µL nuclease-free water]. PCR cycling was performed in Flex Real-Time PCR System (Applied Biosystems, Foster City, CA, USA) using the following conditions: hot start for 10 min at 95 ℃, followed by ten touchdown cycles (15 s at 95 ℃; touchdown at 61 ℃ initially and decreasing by -0.6 ℃ per cycle for 60 s), followed by 28 additional cycles of annealing (15 s at 95 ℃; 60 s at 55 ℃). An extension step was unnecessary as amplicons are usually less than 120 bp long.

**References**

Abrusan, G., Grundmann, N., DeMester, L. and Makalowski, W. (2009) TEclass--a tool for automated classification of unknown eukaryotic transposable elements. *Bioinformatics*, **25**, 1329-1330.

Alexander, D.H., Novembre, J. and Lange, K. (2009) Fast model-based estimation of ancestry in unrelated individuals. *Genome res*. **19**, 1655-1664.

Bao, Z. and Eddy, S.R. (2002) Automated de novo identification of repeat sequence families in sequenced genomes. *Genome res.* **12**, 1269-1276.

Bolger, A.M., Lohse, M. and Usadel, B. (2014) Trimmomatic: a flexible trimmer for Illumina sequence data. *Bioinformatics*, **30**, 2114-2120.

Brian J. Haas, Arthur L. Delcher, Stephen M. Mount, Jennifer R. Wortman, Roger K. Smith Jr, Linda I. Hannick, Rama Maiti, Catherine M. Ronning, Douglas B. Rusch, Christopher D. Town, Steven L. Salzberg and White, O. (2003) Improving the *Arabidopsis* genome annotation using maximal transcript alignment assemblies. *Nucleic Acids res*. **21**, 5654-5666.

Browning, Brian L. and Browning, Sharon R. (2016) Genotype Imputation with Millions of Reference Samples. *Am. J. Hum. Genet.* **98**, 116-126.

Conesa, A., Gotz, S., Garcia-Gomez, J.M., Terol, J., Talon, M. and Robles, M. (2005) Blast2GO: a universal tool for annotation, visualization and analysis in functional genomics research. *Bioinformatics*, **21**, 3674-3676.

Danecek, P., Auton, A., Abecasis, G., Albers, C., Banks, E., DePristo, M., Handsaker, R., Lunter, G., Marth, G., Sherry, S. and McVean, G. (2011) The variant call format and VCFTools. *Bioinformatics*, **27**, 2156-2158.

Edgar, R.C. (2004) MUSCLE: multiple sequence alignment with high accuracy and high throughput. *Nucleic Acids res*. **32**, 1792-1797.

Haas, B.J., Papanicolaou, A., Yassour, M., Grabherr, M., Blood, P.D., Bowden, J., Couger, M.B., Eccles, D., Li, B., Lieber, M., MacManes, M.D., Ott, M., Orvis, J., Pochet, N., Strozzi, F., Weeks, N., Westerman, R., William, T., Dewey, C.N., Henschel, R., LeDuc, R.D., Friedman, N. and Regev, A. (2013) De novo transcript sequence reconstruction from RNA-seq using the Trinity platform for reference generation and analysis. *Nat. Protoc*. **8**, 1494-1512.

Kircher, M., Stenzel, U. and Kelso, J. (2009) Improved base calling for the Illumina Genome Analyzer using machine learning strategies. *Genome Biol*. **10**, R83.

Koren, S., Walenz, B.P., Berlin, K., Miller, J.R., Bergman, N.H. and Phillippy, A.M. (2017) Canu: scalable and accurate long-read assembly via adaptive k-mer weighting and repeat separation. *Genome res.* **27**, 722-736.

Korf, I. (2004) Gene finding in novel genomes. *BMC bioinformatics*, **5**, 59.

Korneliussen, T.S., Albrechtsen, A. and Nielsen, R. (2014) ANGSD: analysis of next generation sequencing data. *BMC Bioinformatics*, **15**, 356.

Langmead, B. and Salzberg, S.L. (2012) Fast gapped-read alignment with Bowtie 2. *Nat. Methods*, **9**, 357-359.

Lee, T.H., Guo, H., Wang, X.Y., Kim, C. and Paterson, A.H. (2014) SNPhylo: a pipeline to construct a phylogenetic tree from huge SNP data. *BMC Genomics*, **15**, 162.

Li, B. and Dewey, C.N. (2011) RSEM: accurate transcript quantification from RNA-Seq data with or without a reference genome. *BMC Bioinformatics*, **12**, 323.

Li, H., Handsaker, B., Wysoker, A., Fennell, T., Ruan, J., Homer, N., Marth, G., Abecasis, G., Durbin, R. and Genome Project Data Processing, S. (2009) The Sequence Alignment/Map format and SAMtools. *Bioinformatics*, **25**, 2078-2079.

Liu, X. and Fu, Y.X. (2015) Exploring population size changes using SNP frequency spectra. *Nat. Genet.* **47**, 555-559.

Lomsadze, A., Ter-Hovhannisyan, V., Chernoff, Y.O. and Borodovsky, M. (2005) Gene identification in novel eukaryotic genomes by self-training algorithm. *Nucleic Acids Res.* **33**, 6494-6506.

McKenna, A., Hanna, M., Banks, E., Sivachenko, A., Cibulskis, K., Kernytsky, A., Garimella, K., Altshuler, D., Gabriel, S., Daly, M. and DePristo, M.A. (2010) The Genome Analysis Toolkit: a MapReduce framework for analyzing next-generation DNA sequencing data. *Genome Res.* **20**, 1297-1303.

Paritosh, K., Yadava, S.K., Singh, P., Bhayana, L., Mukhopadhyay, A., Gupta, V., Bisht, N.C., Zhang, J., Kudrna, D.A., Copetti, D., Wing, R.A., Reddy Lachagari, V.B., Pradhan, A.K. and Pental, D. (2020) A chromosome-scale assembly of allotetraploid Brassica juncea (AABB) elucidates comparative architecture of the A and B genomes. *Plant Biotechnol. J.* 1-13.

Pavlidis, P., Zivkovic, D., Stamatakis, A. and Alachiotis, N. (2013) SweeD: likelihood-based detection of selective sweeps in thousands of genomes. *Mol. Biol. Evol.* **30**, 2224-2234.

Price, A.L., Jones, N.C. and Pevzner, P.A. (2005) De novo identification of repeat families in large genomes. *Bioinformatics*, **21 Suppl 1**, i351-i358.

Purcell, S., Neale, B., Todd-Brown, K., Thomas, L., Ferreira, M.A., Bender, D., Maller, J., Sklar, P., de Bakker, P.I., Daly, M.J. and Sham, P.C. (2007) PLINK: a tool set for whole-genome association and population-based linkage analyses. *Am. J. Hum. Genet.* **81**, 559-575.

Rosenberg, N.A. (2004) DISTRUCT: A program for the graphical display of population structure. *Mol. Ecol. Resour.* **4**, 137-138.

Schliep, K.P. (2011) phangorn: phylogenetic analysis in R. *Bioinformatics*, **27**, 592-593.

Simao, F.A., Waterhouse, R.M., Ioannidis, P., Kriventseva, E.V. and Zdobnov, E.M. (2015) BUSCO: assessing genome assembly and annotation completeness with single-copy orthologs. *Bioinformatics*, **31**, 3210-3212.

Stanke, M., Schoffmann, O., Morgenstern, B. and Waack, S. (2006) Gene prediction in eukaryotes with a generalized hidden Markov model that uses hints from external sources. *BMC bioinformatics*, **7**, 62.

Tamura, K., Peterson, D., Peterson, N., Stecher, G., Nei, M. and Kumar, S. (2011) MEGA5: molecular evolutionary genetics analysis using maximum likelihood, evolutionary distance, and maximum parsimony methods. *Mol. Biol. Evol.* **28**, 2731-2739.

Xie, T., Zheng, J.F., Liu, S., Peng, C., Zhou, Y.M., Yang, Q.Y. and Zhang, H.Y. (2015) De novo plant genome assembly based on chromatin interactions: a case study of *Arabidopsis thaliana*. *Mol. Plant*, **8**, 489-492.
